# Functional Magnetic Resonance Imaging (fMRI) Signatures of Progression and Phenoconversion in Prodromal Synucleinopathies

**DOI:** 10.1002/mds.70025

**Published:** 2025-09-01

**Authors:** Lachlan Churchill, Anna Ignatavicius, Ajay Konuri, Jack Anderson, Natasha Taylor, Simon J.G. Lewis, Elie Matar

**Affiliations:** ^1^ Central Clinical School Faculty of Medicine and Health, University of Sydney Sydney New South Wales Australia; ^2^ Macquarie Medical School and Macquarie University, Centre for Parkinson's Disease Research, Faculty of Medicine, Health and Human Sciences Macquarie University Sydney New South Wales Australia; ^3^ Centre for Integrated Research and Understanding of Sleep (CIRUS), Woolcock Institute for Medical Research Sydney New South Wales Australia; ^4^ School of Medical Sciences Faculty of Medicine and Health, University of Sydney Sydney New South Wales Australia; ^5^ Department of Neurology Royal Prince Alfred Hospital Camperdown New South Wales Australia

**Keywords:** REM sleep behavior disorder, synucleinopathies, Parkinson's disease, magnetic resonance imaging, neuroimaging

## Abstract

**Background:**

Isolated rapid eye movement (REM) sleep behavior disorder (iRBD) is a prodromal manifestation of synucleinopathies and provides a critical window to identify early markers of progression to Parkinson's disease (PD) and dementia with Lewy bodies (DLB). Time‐averaged (static) and time‐varying (dynamic) functional connectivity between large‐scale brain networks may sensitively capture early pathophysiological changes and offer prognostic value beyond structural imaging.

**Objectives:**

To use functional magnetic resonance imaging (fMRI) on a longitudinal iRBD cohort to assess alterations in static and dynamic functional connectivity and explore their relationship with disease conversion and regional neurotransmitter density.

**Methods:**

Static and dynamic resting state fMRI and clinical testing were acquired from 41 iRBD participants and 38 healthy controls, with 21 iRBD participants undergoing repeated scanning.

**Results:**

Cross‐sectional analysis revealed reduced static connectivity within the visual network and a shift toward a more segregated functional architecture in iRBD. Longitudinally, a further increase in segregation was observed, characterized by heightened modularity and reduced intermodular connectivity. These changes were accompanied by static connectivity disruptions in somatomotor and attentional networks, particularly pronounced in patients who converted to DLB. Regions showing the greatest connectivity decline overlapped with areas rich in cholinergic and noradrenergic transporters, suggesting early neuromodulatory dysfunction as a potential driver.

**Conclusions:**

Our findings reveal progressive functional segregation and widespread disrupted static connectivity of resting‐state networks in iRBD. These results identify imaging biomarkers of disease progression, describe likely neurotransmitter associations, and support the implementation of fMRI as a sensitive tool for detecting early neurobiological signatures of synucleinopathies. © 2025 The Author(s). *Movement Disorders* published by Wiley Periodicals LLC on behalf of International Parkinson and Movement Disorder Society.

Isolated rapid eye movement (REM) sleep behavior disorder (iRBD) is a parasomnia characterized by the loss of skeletal muscle atonia during REM sleep, resulting in dream enactment behaviors.^1^ It is now widely considered as a prodromal α‐synucleinopathy^2^ and is integral to current pathophysiological models of Parkinson's disease (PD) progression.^3^, ^4^

Neuropathological and imaging evidence in PD and dementia with Lewy bodies (DLB) suggest that RBD may reflect early dysfunction of the brainstem regions regulating REM sleep atonia,^5^, ^6^, ^7^, ^8^ which is usually followed by early pathological involvement of key neuromodulatory nuclei including the noradrenergic locus coeruleus (LC) and cholinergic nucleus basalis of Meynert (NBM), both of which play a critical role in regulating cortical arousal and cognitive function.^5^, ^9^, ^10^ Despite emphasis on nigrostriatal dopaminergic degeneration in these conditions,^11^, ^12^, ^13^, ^14^ positron emission tomography (PET) imaging studies in iRBD have revealed widespread cholinergic^15^, ^16^, ^17^ and noradrenergic^18^, ^19^, ^20^ cortical denervation in iRBD, despite preserved neuronal integrity in the LC and NBM.^10^ These early neurochemical disruptions likely perturb large‐scale functional networks,^21^ and investigating their sequential involvement could offer critical insights into mechanisms of disease progression and symptom emergence.

Functional MRI (fMRI) is optimally positioned to track the integrity of these functional connections given its safety, accessibility, and capacity to detect network‐level alterations.^22^ Converging fMRI studies have identified disrupted functional connectivity in posterior brain networks and reduced basal–frontal connectivity as key functional signatures in iRBD (for a review see Churchill et al.^23^). There is also growing recognition of the utility of dynamic functional connectivity – an fMRI technique that incorporates the temporal dimension of functional imaging and captures moment‐to‐moment fluctuations in brain organization. Unlike static connectivity, which reflects average correlations over time, dynamic connectivity allows the identification of shifting ‘states’ of high (integrated) and low (segregated) global interconnectivity,^24^ the balance of which has been shown to reflect the integrity of the cholinergic and noradrenergic neurotransmitter systems.^25^, ^26^ Previous work has highlighted disruption in dynamic connectivity across brain networks in advanced PD and DLB, with less integrated and modular (segregated) cortical architecture associating with cognitive impairment and cognitive fluctuations.^27^, ^28^ Such findings suggest a pathological shift towards excessive functional segregation may serve as a biomarker of impending cognitive dysfunction progression across the Lewy body disease spectrum, including possibly in the prodromal stages.^29^, ^30^, ^31^, ^32^, ^33^

Despite the potential of functional neuroimaging to track Lewy body diseases at the earliest stages, longitudinal studies in iRBD remain limited.^34^ To address this gap, we applied fMRI to a well‐phenotyped iRBD cohort including a longitudinal subset of participants with repeated visits. We aimed to examine static and dynamic functional connectivity signatures in iRBD compared with controls and determine their clinical correlates; assess longitudinal changes within the iRBD group and evaluate whether baseline functional signatures predict future disease conversion to PD or DLB; and investigate the neurobiological underpinnings of these functional changes leveraging the known spatial dopaminergic, cholinergic, and noradrenergic receptors and transporters. Building on prior evidence of disrupted occipital and subcortical connectivity in prodromal α‐synucleinopathies,^35^, ^36^, ^37^, ^38^ we hypothesized that iRBD patients would show similar reductions in functional connectivity within these regions compared with controls. We further hypothesized that dynamic connectivity would reveal heightened cortical segregation in iRBD patients at baseline, with further increases over time, especially in patients who developed dementia.

## 1 Methods

### 1.1 Participants

Forty‐one participants with video polysomnography‐confirmed iRBD were recruited from the Brain and Mind Centre, University of Sydney. Diagnosis of iRBD was confirmed by neurologists and sleep specialists according to the International Classification of Sleep Disorders (ICSD‐III, American Academy of Sleep Medicine [AASM]).^39^

Alongside the iRBD cohort, 38 age‐matched healthy controls were recruited and underwent comprehensive clinical and neuropsychological tests (Supplementary Methods). Repeat visits were conducted on 21 iRBD participants, with an average follow up time of 2.75 years. Of the 21, 6 returned for a third visit with an average of 4.7 years between the initial and last visit. Eight of the participants transitioned to neurodegenerative disorders before their final scan: four to PD and four to DLB, respectively. All participants provided consent to involvement in the research, which was approved by the University of Sydney Human Research Ethics Committee.

### 1.2 MRI Acquisition and Preprocessing

Imaging included acquiring a whole brain T1‐weighted image and T2*‐weighted functional images on a General Electric 3 T system. Preprocessing of the fMRI data was conducted using fMRIprep (https://fmriprep.org/)^40^ followed by denoising using fMRIdenoise (https://github.com/compneuro-ncu/fmridenoise) and parcellation using a combined atlas incorporating the Schaefer 400 cortical regions parcellation, Tian's 54 subcortical region parcellation, and a probabilistic anatomical segmentation of the NBM and LC derived from postmortem tissue.^41^, ^42^, ^43^, ^44^ Further information regarding imaging acquisition and preprocessing is detailed in the Supporting Information Methods.

### 1.3 Resting State Network‐Based Static Functional Connectivity

The 400 cortical regions were initially assigned to the 17 resting state networks developed by Yeo et al.,^45^ which were subsequently grouped into broader eight resting state networks to allow for more meaningful, large‐scale network comparisons.^42^, ^45^ These networks are based on data‐driven parcellations and have become a widely used standard in resting‐state fMRI studies, providing a biologically grounded and reproducible framework for functional connectivity analysis. Whole‐cortex (global) functional connectivity was computed by averaging the pairwise functional connectivity values across all 400 cortical nodes. Network‐to‐cortex functional connectivity was calculated by first computing the connectivity between each node within a given network and all 400 cortical nodes, followed by averaging these values across all nodes within the network. Internetwork functional connectivity was derived by averaging the connectivity values between all node pairs belonging to two distinct networks. To assess the subcortical contribution of regions associated with cholinergic, noradrenergic, and dopaminergic neurotransmission, we first extracted the functional connectivity matrices from bilateral NBM, LC, and the basal ganglia (BG), comprised of the caudate and putamen.^41^ For each subcortical region of interest (ROI), we calculated the average functional connectivity to nodes within each of the eight resting‐state networks.

### 1.4 Dynamic Functional Connectivity Analysis

#### 1.4.1 Temporal Correlations of Functional Configurations

As an initial measure of functional dynamics, we examined temporal differences in brain state configurations, categorized into two distinct metrics: local similarity (S_L_) and global similarity (S_G_). Pearson's correlation of BOLD activity across the whole brain computed across each time epoch (TR) of the scan.^28^

Local similarity refers to the correlation of neural activity within each parcel across consecutive timepoints during a scan. Higher local similarity values indicate more consistent neuronal configuration patterns, reflecting a more stationary brain state over the duration of the scan.

Unlike local similarity, which focuses on consecutive time points, global similarity evaluates regional correlations across the entire scan. Higher global similarity values indicate lower variability in functional configurations.

#### 1.4.2 Temporal Derivatives and Community Assignment

To accurately incorporate the time‐resolved community structure of functional connectivity over the scan we initially calculated the multiplication of temporal derivatives for each time series with a sliding window of 15 TRs (code available: https://github.com/lachychurchill/coupling_integration/).^24^ Next, we implemented Louvain community assignment to classify nodes into modules throughout the course of the scan and the subsequent analysis using the Brain Connectivity Toolbox^46^ (https://sites.google.com/site/bctnet/). Within‐module connectivity was estimated by calculating the module degree z‐score (W_T_) for each parcel.^47^ Higher module degree z‐scores indicate that a parcel is more strongly connected with its own module and plays a central role in local (intramodular) processing.

Between‐module connectivity was calculated using the participation coefficient (B_T_) and provides insight on the extent to which a region is connected to other wide‐ranging modules compared with its intramodular connectivity. Participation scores that are closer to 1 represent a wide range of intermodular connectivity compared with those that are closer to 0.

Based on this characterization, we clustered brain states into two categories (*k* = 2): highly integrated states (high B_T_ and low W_T_) and highly segregated states (low B_T_ and high W_T_) across the scan for each patient, as previously described.^48^ We then analyzed brain state transitions, and the characteristics associated with shifts between integrated and segregated states.

The time‐resolved modularity score (Q) was also calculated in each participant, indicating the number of distinct modules the brain could be divided into at that time. A higher modularity indicates that the cortical organization is comprised of a larger number of distinct subnetworks.^49^ Further information regarding dynamic functional connectivity measures can be found in the Supporting Information Methods.

### 1.5 Neurotransmitter Receptor Density Mapping

To translate the neurobiological basis into functional connectivity measures, we used the recently developed neurotransmitter receptor density mapping approach (code available: https://github.com/netneurolab/hansen_receptors).^50^ Functional connectivity and participation coefficients for the 400 Schaefer regions were extracted and analyzed statistically to assess disparities between groups both cross sectionally and longitudinally. The test statistics for each region were then correlated with the cholinergic, dopaminergic, and noradrenergic receptor densities using Spearman's rank correlation.

### 1.6 Statistical Analysis

All statistical analyses were performed using MATLAB R2023b (The MathWorks, Inc., Massachusetts, USA). For the cross‐sectional analysis, demographic variables were compared using chi‐squared tests for binary data and independent samples *t*‐tests for continuous data. Static and dynamic functional connectivity measures were analyzed using general linear models, incorporating age, sex, and education as covariates. Correlations between functional connectivity measures and clinical variables were assessed within the iRBD cohort using Spearman rank correlation analysis. For the longitudinal analysis of iRBD participants, variables were examined using a linear mixed‐effects model with time (measured in months) as a continuous variable of interest with age, sex, and education as covariates.

A Cox proportional hazards model was used to assess the influence of functional connectivity measures on the relative risk of future phenoconversion. Longitudinal clinical follow‐up data were available for all 41 iRBD patients initially scanned and phenotyped at baseline, of whom 10 converted to PD and 7 to DLB. Two separate survival models were examined: the first assessed conversion to any α‐synucleinopathy (PD or DLB), and the second focused specifically on conversion to DLB. A robust description of statistical models is outlined in the Supporting Information Methods.

## 2 Results

### 2.1 Participants

Baseline sample characteristics are summarized in Table 1. There were no notable differences in age, education, or medication use between groups. There were no significant differences between iRBD participants and controls on sleep‐related questionnaires. Cognitive performance, specifically in the Montreal Cognitive Assessment (MoCA) and Trail Making Test Part B (TMT‐B), was significantly impaired in the iRBD group (*P* = 0.012 and *P* = 0.037, respectively). iRBD participants exhibited a trending decline in Stroop inhibition/switching scores over time (*P* = 0.057), while other cognitive and clinical scores remained stable (Table S1). Demographic characteristics stratified by sex are provided in Table S2.

**TABLE 1:** Demographics and clinical characteristics of isolated rapid eye movement sleep behavior disorder patients and controls

Clinical variable	Controls (N = 38)	iRBD (N = 41)	*P‐*value
Age (years)	66.95 (7.55)	65.93 (6.68)	0.516
Education	14.24 (2.79)	13.15 (3.01)	0.095
Disease duration (years)	–	1.94 (2.20)	–
Disease conversion	–	–	–
Sex (male:female)	17: 21	34: 7	**<0.001**
SSRI (% on)	7.9%	7.3%	0.999
SNRI (% on)	2.6%	0%	0.969
Benzodiazepines (% on)	5.3%	9.8%	0.742
Questionnaires			
SCOPA Sleep Night	4.06 (3.62)	2.66 (3.25)	0.148
SCOPA Sleep Day	2.28 (2.11)	2.66 (3.64)	0.692
Epworth Sleepiness Scale	5.23 (3.20)	7.06 (5.54)	0.148
RBDSQ	2.59 (2.04)	7.52 (3.23)	**<0.001**
HADS Anxiety	2.64 (2.16)	3.13 (3.41)	0.648
HADS Depression	1.76 (1.81)	2.55 (3.44)	0.704
MDS‐UPDRS Question 1.2 (Visual Hallucination Score)	0 (0)	0.06 (0.24)	0.144
Cognitive measures			
Montreal Cognitive Assessment	28.03 (2.04)	26.68 (2.58)	**0.012**
Trail Making Test Part B	0.59 (0.63)	0.12 (1.10)	**0.037**
Digit Span Backwards	12.50 (2.76)	11.77 (2.94)	0.327
Stroop Inhibition	11.68 (3.09)	11.11 (2.6)	0.467
Stroop Inhibition/Switching	12.18 (2.48)	11.31 (2.8)	0.248
Sensory/motor			
Sniffin Sticks	10.13 (1.36)	7.10 (2.96)	**<0.001**
MDS‐UPDRS‐III	1.26 (1.81)	9.17 (8.32)	**<0.001**

*Note*: Values are displayed as mean (standard deviation). *P‐*values were calculated using nonparametric permutation testing and chi‐squared tests where applicable. Bold type indicates statistical significance of *p* < 0.05.

Abbreviations: iRBD, isolated rapid eye movement sleep behavior disorder; SSRI, selective serotonin reuptake inhibitor; SNRI, serotonin–norepinephrine reuptake inhibitor; SCOPA, Scales for Outcomes in Parkinson's Disease; RBDSQ, REM Sleep Behavior Disorder (RBD) Screening Questionnaire; HADS, Hospital Anxiety and Depression Scale; MDS‐UPDRS‐III, Movement Disorder Society Unified Parkinson's Disease Rating Scale‐Part III.

### 2.2 Altered Inter‐Network Static and Dynamic Functional Connectivity in iRBD

**FIG. 1:**
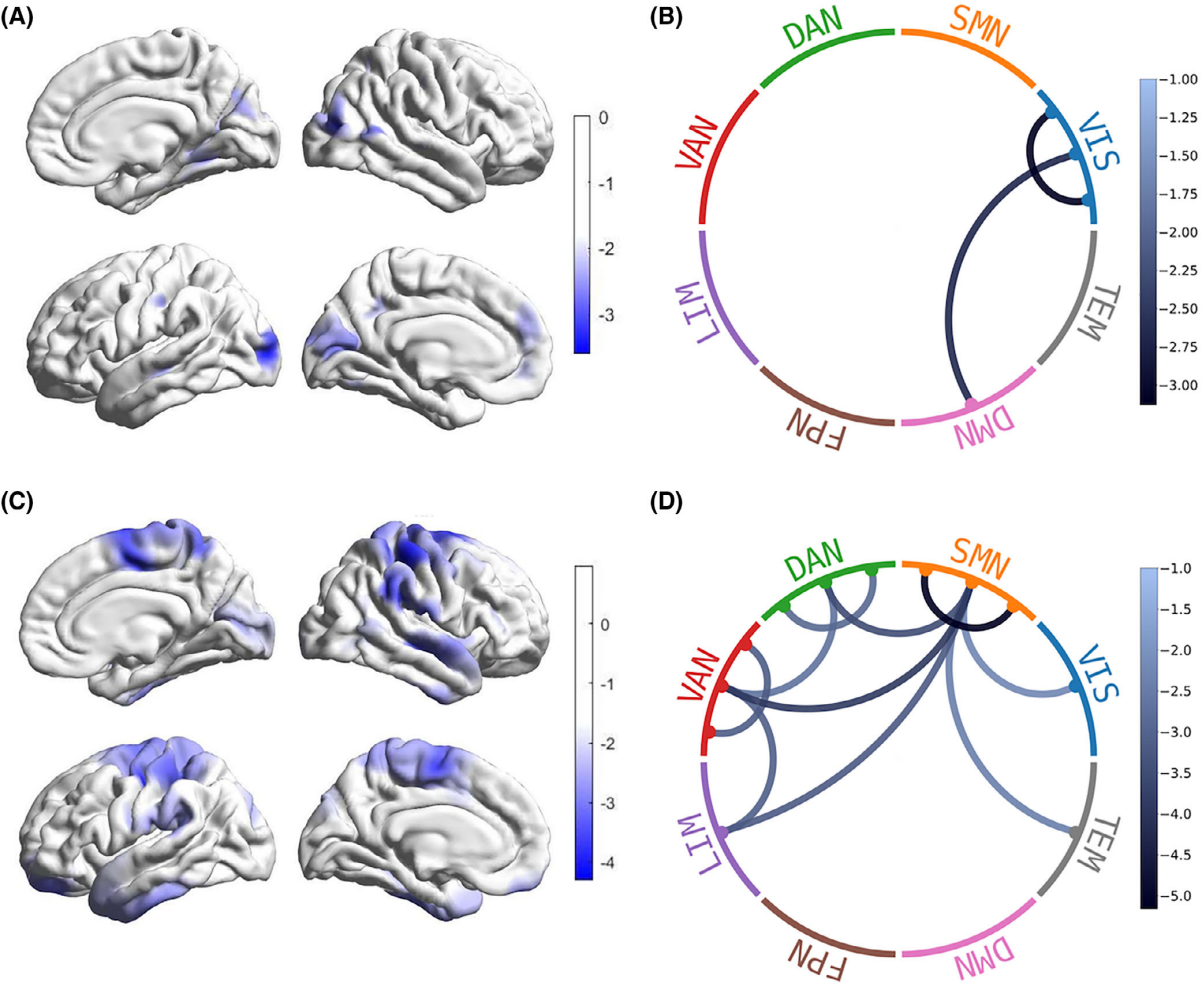
Reduced cross‐sectional and longitudinal static functional connectivity of resting state networks. (A) A trend of reduced average functional connectivity in regions within the visual network in isolated rapid eye movement (REM) sleep behavior disorder (iRBD) compared with healthy controls. The scale bar denotes the test statistic for average functional connectivity across all 400 regions derived from a general linear model comparing iRBD and control groups with age, sex, and education as covariates. (B) Significant reductions in the average static functional connectivity intrinsically within the visual network and between the visual network and the default mode network in iRBD compared with healthy controls. The scale bars denote the test statistic derived from the same previously described general linear model.^51^ (C) Reduced average functional connectivity between the cortex and regions in the somatomotor, dorsal attentional, and ventral attentional networks in iRBD participants over time. The scale bar denotes the test statistic derived from a linear mixed‐effects model with time as the variable of interest and age, sex, and education as covariates. (D) Significant reductions in the average static functional connectivity intrinsically within the somatomotor, dorsal attentional. and ventral attentional networks in iRBD participants over time. Somatomotor network functional connectivity was reduced to the visual, dorsal attentional, ventral attentional, limbic, and temporal networks. Ventral attentional network functional connectivity was reduced to the dorsal attentional and limbic networks. The scale bars denote the test statistic derived from the same previously described linear mixed‐effects model.^51^ DAN, dorsal attentional network; SMN, somatomotor network; VAN, ventral attentional network; VIS, visual network; LIM, limbic network; TEM, temporal network; FPN, frontoparietal network; DMN, default mode network. [Color figure can be viewed at wileyonlinelibrary.com]

There were no significant differences in whole‐cortex functional connectivity (*p*_*FDR*_ = 0.124) (Table S3). Network‐to‐cortex analysis revealed a trend towards decreased functional connectivity in the visual network (uncorrected two‐sided *P* = 0.008, corrected *p*_*FDR*_ = 0.060). Inter‐network connectivity analysis revealed reduced functional connectivity between nodes of the visual network (reduced self‐connectivity) and between the visual and the default mode networks (both values *p*_*FDR*_ = 0.020) (Fig. 1A, B; Table S4). Connectivity was not altered between the a priori selected subcortical seeds (BG, NBM, and LC) and the resting state networks (Table S5).

For dynamic functional connectivity measures, participants with iRBD exhibited significantly increased modularity compared with controls (*P* = 0.039), alongside a reduced local similarity (*P* = 0.042) (Table S6). Despite iRBD patients exhibiting a more modular brain state, there were no significant differences in between‐ or within‐module connectivity, and segregated state characterization relative to controls (Table S7). There was a trend of reduced local similarity (rho = 0.36, uncorrected *P* = 0.030) and increase in state transitions (rho = −0.42, uncorrected *P* = 0.008) being correlated with worsened TMT‐B scores; however, this did not reach significance after false discovery rate (FDR) correction (Table S8).

### 2.3 Whole‐Cortex and Internetwork Connectivity is Reduced Over Time in iRBD

Whole‐cortex functional connectivity significantly decreased over time in iRBD participants (*P* = 0.013). Network to whole‐cortex functional connectivity was reduced in the somatomotor (*p*_*FDR*_ = 0.005), dorsal attentional (*p*_*FDR*_ = 0.028), and ventral attentional networks (*p*_*FDR*_ = 0.017) (Fig. 1C). Internetwork connectivity showed reduced somatomotor network connectivity with the visual, dorsal attentional, ventral attentional, limbic, and temporal networks. Connectivity was also reduced between the ventral attentional network and the dorsal attentional and limbic networks (Fig. 1D; Table S9). No significant differences were observed in subcortical functional connectivity (Table S10).

**FIG. 2:**
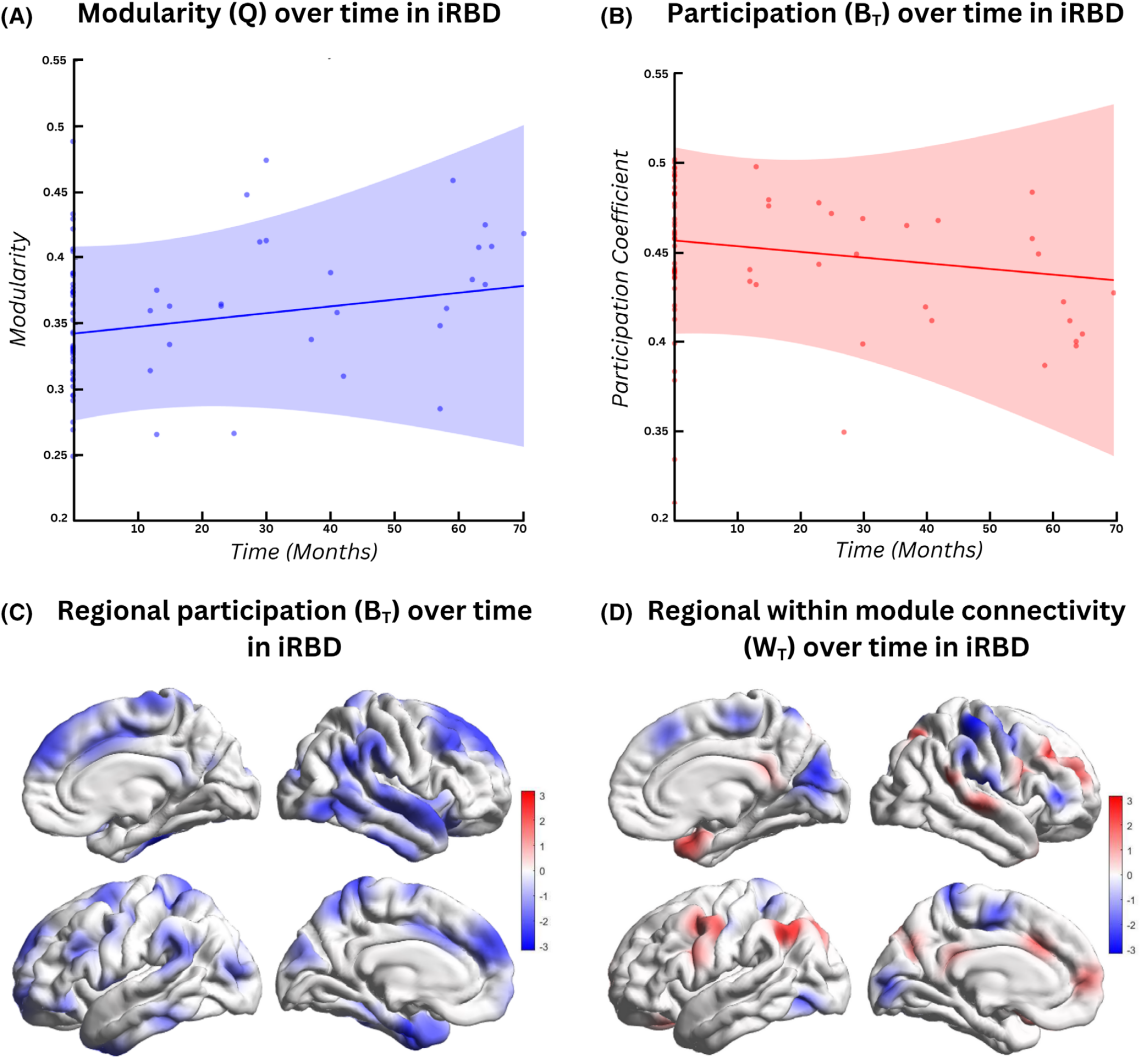
Longitudinal cortical‐ and network‐based dynamic functional connectivity changes. (A) Increased whole‐cortex modularity was observed in isolated rapid eye movement (REM) sleep behavior disorder (iRBD) patients over time. (B) Decreased whole‐brain participation (between module connectivity) was demonstrated in iRBD patients over time. (C) Significantly reduced average participation was demonstrated in nodes comprising the dorsal, ventral, limbic, and temporal networks over time in iRBD patients. The scale bar denotes the test statistic for all 400 regions participation coefficients derived from a linear mixed‐effects model with time as the variable of interest and age, sex, and education as covariates. (D) Significantly reduced average module degree z‐score (within‐module connectivity) was demonstrated in nodes comprising the somatomotor network and increased module degree z‐score in nodes comprising the frontoparietal network over time in iRBD patients. The scale bar denotes the test statistic for all 400 regions module degree z‐score derived from the previously described model. [Color figure can be viewed at wileyonlinelibrary.com]

In relation to the macroscale dynamic changes, participants with iRBD demonstrated a significant increase in modularity over time (*P* = 0.023) (Fig. 2A). Additionally, a decrease in whole‐cortex participation (B_T_) was observed (*P* = 0.049) (Fig. 2B; Table S11). Notably, this was localized to the dorsal attentional, ventral attentional, limbic, and temporal networks (*P* < 0.05) (Fig. 2C). Within‐module degree z‐score (W_T_) was reduced in the somatomotor network (*P* = 0.006) but increased in the frontoparietal network (*P* = 0.001) in iRBD participants over time (Fig. 2D). No significant differences were found in dwell time or transitions between integrated and segregated states. Both local and global similarity scores remained unchanged. Associations between changes in functional connectivity measures and changes in cognitive and motor function over time are further detailed in Table S12.

### 2.4 Baseline Clinical and Imaging Predictors of Phenoconversion in iRBD

Baseline MoCA and TMT‐B scores were significantly associated with disease conversion. Specifically, for every 1 point decrease in MoCA, the risk (hazard) of conversion increased by 44% (hazard ratio [HR] = 1.44, 95% confidence interval [CI]: 1.14–1.81, *P* = 0.002), and the risk of conversion to DLB increased by 76% (HR = 1.76, CI: 1.18–2.63, *P* = 0.006). Similarly, each 1 standard deviation (SD) decrease in z‐scored TMT‐B performance was associated with a doubling of the conversion risk (HR = 2.00, CI: 1.12–3.56, *P* = 0.019), and a nearly three‐fold increased hazard of conversion to DLB (HR = 2.98, CI: 1.13–7.82, *P* = 0.026).

Functional connectivity (z‐scored) between the LC and visual network was also significantly associated with an increased risk of disease progression. A 1 SD decrease in this connectivity measure was associated with a 66% increased risk of conversion (HR = 1.66, CI: 1.00–2.76, *P* = 0.048), and a 126% increased risk of conversion to DLB (HR = 2.26, CI: 1.13–6.16, *P* = 0.025). A full summary of the survival analysis results is provided in Table S13.

### 2.5 Longitudinal Functional Connectivity Changes Predict Conversion to Dementia in iRBD

**FIG. 3:**
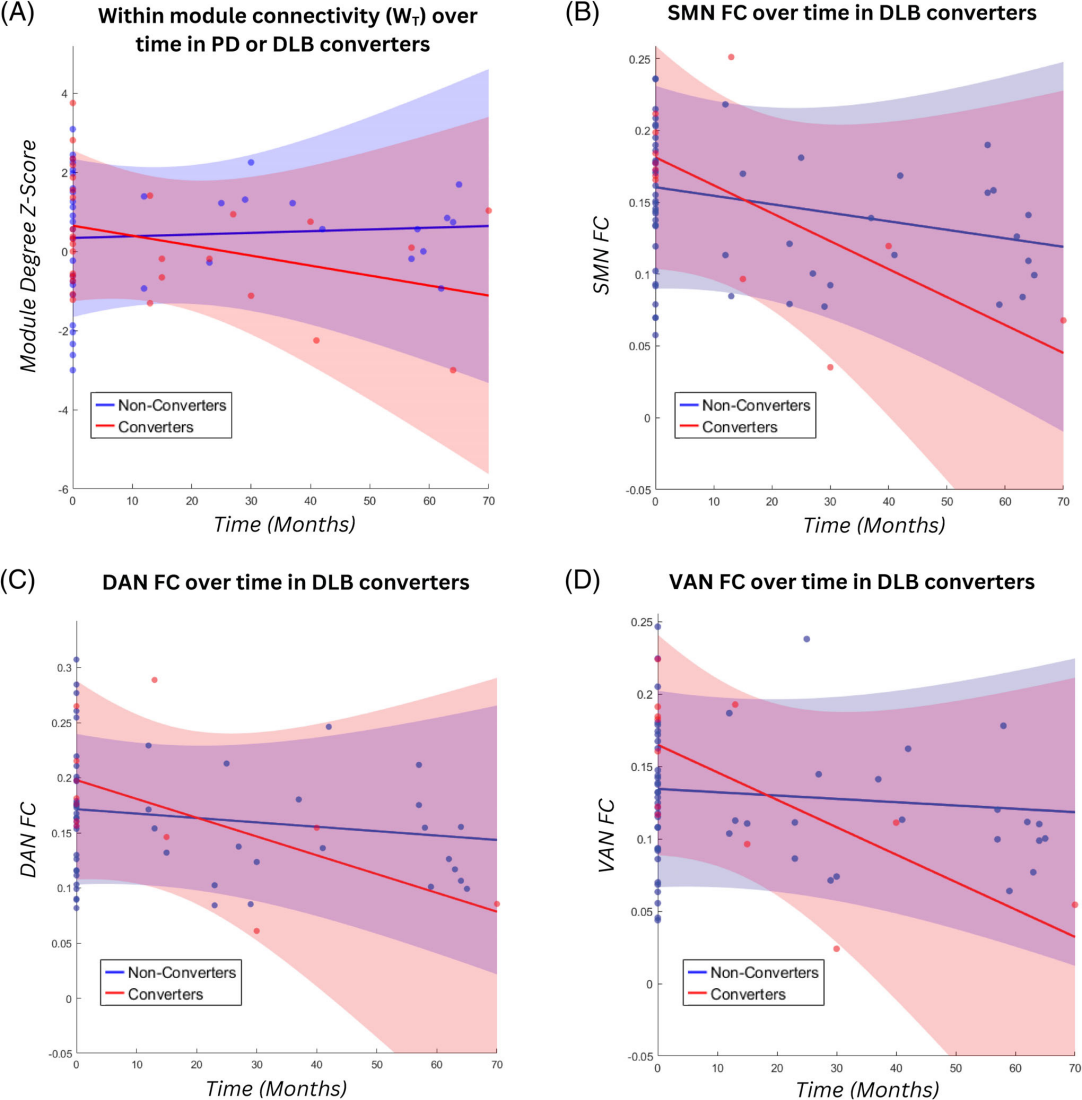
Progression of static and dynamic functional connectivity measures in Parkinson's disease (PD) and dementia with Lewy bodies (DLB) converters. (A) Participants who converted to either PD or DLB had a greater reduction in within‐module connectivity over time compared with non‐converters. Participants who converted to dementia had a greater reduction in (B) somatomotor network, (C) dorsal attentional network, and (D) ventral attentional network functional connectivity over time compared with those who did not convert to dementia. SMN, somatomotor network; FC, functional connectivity; DAN, dorsal attentional network; VAN, ventral attentional network. [Color figure can be viewed at wileyonlinelibrary.com]

A reduction in module degree z‐score was the only measure, static or dynamic, that showed a significantly different trajectory over time in participants who converted to either PD or DLB (*P* = 0.025; Fig. 3A). Whole‐cortex functional connectivity (*P* = 0.010), as well as network‐to‐cortex functional connectivity in the somatomotor (*P* = 0.023), dorsal attentional (*P* = 0.048), and ventral attentional (*P* = 0.001) networks showed significant declines over time in participants who converted to DLB specifically (Table S14; Fig. 3B–D). Longitudinal changes in dynamic functional connectivity measures did not significantly associate with dementia conversion.

### 2.6 Neuromodulatory Contributions to Functional Connectivity Signatures in iRBD

**FIG. 4:**
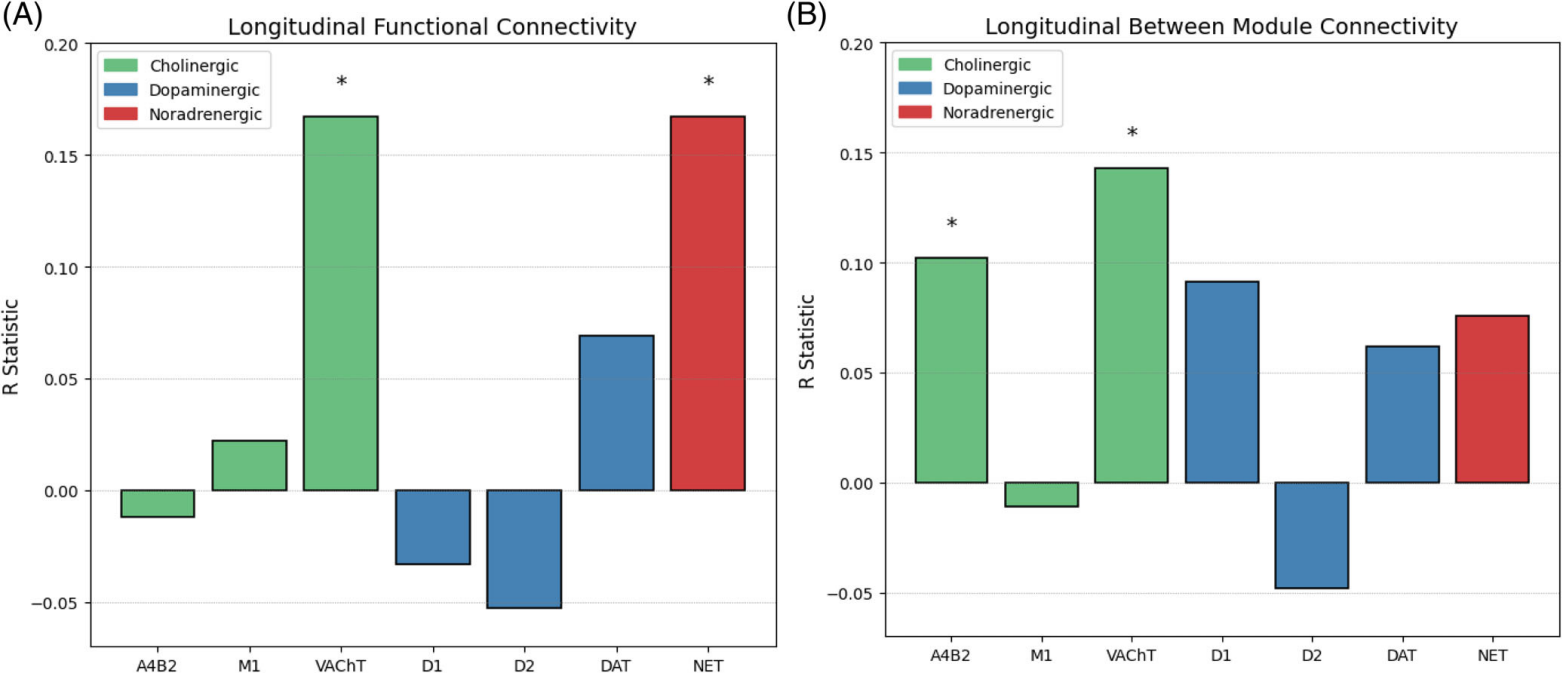
Spearman's rank correlations between longitudinal static and dynamic functional connectivity measures. (A) Correlation between neurotransmitter/gene expression density and the differences between average connectivity in the 400 cortical regions, denoted by the T statistic derived from the general linear model. Regions with higher density of vesicular acetylcholine transporter and noradrenergic were associated with the largest differences of functional connectivity in isolated rapid eye movement (REM) sleep behavior disorder (iRBD) patients over time. (B) Correlation between neurotransmitter/gene expression density and the differences between participation coefficient (integration measure) in the 400 cortical regions, denoted by the T statistic derived from the linear mixed‐effects model. A4B2, alpha‐4 beta‐2 nicotinic acetylcholine receptor; M1, muscarinic acetylcholine receptor M1; VAChT, vesicular acetylcholine transporter; D1, dopamine D1 receptor; D2, dopamine D2 receptor; DAT, dopamine transporter; NET, norepinephrine transporter. [Color figure can be viewed at wileyonlinelibrary.com]

Spearman's rank correlation analysis revealed that larger changes in longitudinal functional connectivity were significantly associated with cortical regions of high vesicular acetylcholine transporter (rho = 0.17, *P* < 0.001) and noradrenergic transporter densities (rho = 0.17, *P* < 0.001). While no significant associations were found between functional connectivity changes and dopamine transporter (DAT) (rho = 0.07, *P* = 0.166) or dopamine 1 (rho = −0.03, *P* = 0.517) and dopamine 2 (rho = −0.05, *P* = 0.291) receptor densities (Fig. 4A).

Significant differences in participation coefficient (B_T_) were also localized to regions with elevated vesicular acetylcholine transporter (rho = 0.14, *P* = 0.004) and α4β2 nicotinic acetylcholine receptor expression (rho = 0.10, *P* = 0.041) (Fig. 4B; Tables S15–S18).

## 3 Discussion

In this study, we have provided a comprehensive and systematic investigation of time‐averaged and, for the first time, longitudinal dynamic functional alterations in iRBD and assessed their relationship to PD and DLB phenoconversion. We observed reduced intrinsic functional connectivity within the visual network in iRBD compared with healthy controls. Additionally, iRBD patients exhibited a more modular and distinct network topology, reflecting a functionally segregated cortical organization that became more pronounced over time. Longitudinal network disruptions within the somatomotor, ventral attentional, and dorsal attentional networks were pronounced in patients who transitioned to DLB and were localized to regions with higher densities of vesicular acetylcholine and noradrenergic transporters. Overall, the findings support the hypothesis that early involvement of neuromodulatory systems contributes to large‐scale network dysfunction and may differentiate phenoconversion trajectories within the synucleinopathy spectrum.

### 3.1 Visual Network Dysfunction Occurs in iRBD and May Predict Phenoconversion

Visuospatial disturbances are frequently reported in the setting of α‐synucleinopathies, and play a central role in the progression of non‐motor symptoms.^52^ Dysfunction within the visual network, primarily composed of regions within the occipital lobe, has been well‐documented in both PD and DLB,^53^, ^54^ where it is associated with cognitive decline and dementia development.^55^, ^56^ Converging evidence from fMRI studies consistently implicate disrupted posterior functional connectivity in iRBD patients compared with controls.^35^, ^57^, ^58^, ^59^, ^60^, ^61^ Other imaging modalities, such as PET and structural MRI, have revealed complementary findings in iRBD, including hypometabolism and cortical thinning in the occipital lobe.^62^, ^63^ In fact, multiple studies have demonstrated occipital hypometabolism being associated with or predictive of disease conversion in iRBD cohorts.^64^, ^65^, ^66^ Despite these associations, the occipital lobe and visual areas exhibit relatively little α‐synuclein deposition, even in the latest stages of the disease. Aligning with these findings, our research demonstrated reduced functional connectivity from LC to visual network as predictive of disease conversion to either PD or DLB. A potential explanation for this selective vulnerability lies in the early pathological burden of the LC in iRBD.^29^, ^67^, ^68^, ^69^, ^70^ Functional disruptions in the LC may manifest earlier and more directly compared with alterations in whole‐brain or higher‐order dynamic connectivity measures. Significant disruption of functional connections of the LC may therefore infer a more advanced degeneration of the LC and a heightened phenoconversion risk. The relatively high proportion of future DLB converters in our cohort may have contributed to the occipital dysfunction observed at the group level. Although visual hallucinations were not observed in our participants, future studies could further investigate occipital dysfunction as a potential biomarker for the later emergence of visual hallucinations.

### 3.2 Macroscopic Brain Dynamic Changes Occur in iRBD

Previous work in PD and DLB has shown that disruptions in dynamic connectivity, particularly increased network segregation and a decline in global brain network efficiency, are associated with disease severity and the emergence of key non‐motor symptoms, including cognitive fluctuations and visual hallucinations.^27^, ^28^, ^71^, ^72^ In alignment with these findings, individuals with iRBD exhibited a pronounced increase in modularity compared with healthy controls, reflecting a greater degree network segregation. To date, one prior cross‐sectional study has applied dynamic fMRI in iRBD, demonstrating that patients spent more time in a segregated brain state relative to controls.^61^ While our results are mostly consistent, our patients did not have an increased dwell time in sparsely connected brain states.^61^ This discrepancy may be explained by a shorter disease duration in our cohort, potentially reflecting an earlier stage. The increase in modularity, accompanied by a decline in intermodular connectivity, was further observed in iRBD patients over time, supporting our hypothesis of a progressively segregated and modular network configuration in iRBD.

### 3.3 Progressive Dysfunction in Attentional Networks Associate with Phenoconversion to Dementia

To date, only one longitudinal fMRI study has been conducted in patients with iRBD, which focused specifically on dysconnectivity of the olfactory cortex.^34^ Our analysis revealed somatomotor, dorsal attentional, and ventral attentional network disruption over time in iRBD participants with more pronounced decline observed in DLB converters. Together, the dorsal and ventral attentional networks orchestrate a coordinated salience response to environmental demands, ensuring efficient cognitive resource allocation. The functional decoupling of these networks appears to be a hallmark of DLB,^73^ where disruptions in attentional processing are suggested to underpin cardinal non‐motor symptomology such as cognitive fluctuations, impaired attention,^74^ and visual hallucinations.^75^, ^76^

### 3.4 Neurochemical Mechanisms Underlying Connectivity Changes in iRBD

Neurotransmitter receptor/transporter density mapping demonstrated that regions with higher densities of vesicular acetylcholine and noradrenergic transporters showed greater decline in longitudinal functional connectivity, implicating these systems in early large‐scale network dysfunction.

Neuromelanin‐sensitive MRI and PET imaging have revealed reduced LC signal intensity and noradrenergic disruption in iRBD similar to findings in PD.^19^, ^20^, ^77^, ^78^ The LC, with its diffuse axonal projections,^31^, ^79^, ^80^ is thought to induce cortical ‘integration’ through phasic firing,^81^ which releases noradrenaline and increases neural gain across multiple brain systems^82^, ^83^, ^84^, ^85^. Converse to phasic firing, a recent optogenetic fMRI study in mice demonstrated that tonic LC activation is linked to a progressively segregated cortical architecture, particularly in transmodal and attentional regions.^86^ The LC has been suggested to be implicated early in PD,^87^ and even as an initiation point of neuropathological spread.^6^, ^7^, ^88^ Early α‐synuclein deposition has been suggested to induce neuronal hyperexcitability^89^ and its deposition in the LC has been proposed to promote a pathological increase of tonic and reduced phasic firing reducing diffuse noradrenaline release.^90^ Thus, early pathological changes in LC may disrupt tonic and phasic firing patterns and have downstream impacts on macroscopic brain dynamics, favoring a more modular and functionally segregated network architecture, as reflected in our findings.

In parallel, early cholinergic dysfunction has previously been demonstrated in molecular imaging studies in iRBD,^16^, ^17^ and more recently in a longitudinal cohort,^15^ further supporting the role of impaired cholinergic neurotransmission in disrupting network dynamics at early stages of the disease.^83^, ^91^ Key nodes in the ventral attentional network, such as the ventral frontal and cingulo‐opercular regions, are densely innervated by highly branched cholinergic neurons, which are particularly vulnerable to degeneration in PD and related disorders.^75^, ^92^, ^93^ These cholinergic projections largely contribute to shaping attentional and perceptual cortical network organization, with disruption to these pathways likely contributing to cognitive impairment.^94^ Participants who subsequently transitioned to DLB exhibited more pronounced disruptions in these networks, reinforcing the hypothesis that cholinergic dysfunction in attentional and sensorimotor networks may represent a critical mechanism underlying dementia conversion in iRBD. Notably, dynamic connectivity metrics, including modularity and participation coefficient, did not distinguish converters from non‐converters. These whole‐brain measures may be less sensitive to the spatially specific disruptions observed in attentional and sensory networks. It is also possible that dynamic connectivity alterations follow a nonlinear trajectory, becoming more pronounced only after critical thresholds of cholinergic or noradrenergic denervation are crossed in more advanced stages of dementia. Larger cohorts with more dementia converters may allow detection of more subtle group differences in these global dynamic measurements.

### 3.5 Limitations and Future Directions

This single‐center study benefits from a well‐characterized cohort and tightly controlled imaging protocols but is limited by a modest sample size. The absence of group differences in sleep questionnaire scores and the relative frequency of PD and DLB conversion may differ from other cohorts but likely reflects the known clinical heterogeneity of iRBD. The lack of change in MOCA scores in our sample at visit 3 may be due to selective attrition as those who did not return for follow‐up after visit 2 had lower average cognitive scores. However, this was accounted for in our longitudinal models, which still suggested a trend toward cognitive decline with time. Larger multicenter longitudinal studies are needed to capture the clinical variability of iRBD and support generalizability to clinical practice. Receptor density analyses were restricted to cortical regions, as subcortical data remain unavailable. Additionally, receptor maps were derived from healthy controls and may not reflect distributions in individuals at risk of α‐synucleinopathy. Participants who had phenoconverted were scanned while on medication, which may have reduced sensitivity to longitudinal effects, though cross‐sectional and prognostic analyses are unlikely to be affected. Medications such as dopaminergic agents, cholinesterase inhibitors, and antidepressants may influence functional connectivity and could have introduced variability. Addressing these limitations will be key to advancing biomarker development in prodromal Lewy body disease.

Functional connectivity provides a sophisticated and potentially sensitive metric for capturing the earliest neural alterations in iRBD. Future studies in larger, multicenter cohorts would allow the establishment of normative thresholds for specific functional metrics (eg, whole‐brain modularity or LC–visual network connectivity), which could be evaluated using receiver operating characteristic analyses and applied at the individual level in clinical settings to assess risk of phenoconversion. In parallel, identifying spatial ‘signatures’ of functional dysconnectivity in regions enriched with cholinergic or noradrenergic receptor density, as shown in our sample, may help infer patterns of early neuromodulatory dysfunction. These functional signatures could support precision medicine approaches by guiding the selection of patients most likely to benefit from neuromodulatory interventions (single or combined cholinergic, dopaminergic, or noradrenergic therapies) and serving as biomarkers to monitor treatment response. Additionally, combining these functional measures with complementary imaging modalities such as diffusion imaging for white matter integrity, PET imaging for synaptic or neurotransmitter function, and neuromelanin‐sensitive MRI for assessing the locus coeruleus and substantia nigra^23^, ^57^, ^95^ would help clarify the mechanistic and temporal order of the neurochemical and anatomical changes observed in early synucleinopathies. Integrating these multimodal imaging findings into a composite biomarker profile may help delineate distinct trajectories of α‐synuclein spread and phenoconversion and inform patient stratification in disease‐modifying trials.

## Author Roles

(1) Research Project: A. Conception, B. Organization, C. Execution; (2) Statistical Analysis: A. Design, B. Execution, C. Review and Critique; (3) Manuscript: A. Writing of the First Draft, B. Review and Critique.

L.C.: 1A, 1B, 1C, 2A, 2B, 3A.

A.I.: 1B, 1C, 2B, 2C, 3B

A.K.: 1B, 1C, 2B, 2C, 3B.

J.A.: 1B, 1C, 2B, 2C, 3B.

N.T.: 1B, 1C, 2B, 2C, 3B

S.J.G.L.: 1B, 2C, 3B.

E.M.: 1A, 1B, 2A, 2C, 3B.

## Disclosures

**Ethical Compliance Statement:** Written informed consent was obtained from all patients, and ethical approval was obtained from the Sydney University Human Research Ethics Committee (HREC No. 2013/HE000945).

**Funding Sources and Conflict of Interest:** L.C. is the recipient of the Bierzonski Burczyk Foundation Postgraduate Research Scholarship. E.M. is supported by a National Health and Medical Research Council Emerging Leadership Fellowship (2008565) and the US Department of Defense Congressionally Directed Medical Research Program Early Investigator Grant (PD220061). S.J.G.L. is supported by a National Health and Medical Research Council Leadership Fellowship (1195830) and has received research funding from The Michael J. Fox Foundation and the Australian Research Council, as well as consulting for Pharmaxis Ltd. The authors declare no competing interests.

## Supporting information


**Supplementary TABLE S1.** Cross‐sectional and longitudinal clinical demographics in isolated rapid eye movement (REM) sleep behavior disorder (iRBD) and controls.
**Supplementary TABLE S2.** Demographics and clinical characteristics of isolated rapid eye movement (REM) sleep behavior disorder (iRBD) patients by sex.
**Supplementary TABLE S3.** Group differences in average functional connectivity between resting state networks and cortical nodes in isolated rapid eye movement (REM) sleep behavior disorder (iRBD) compared with controls.
**Supplementary TABLE S4.** Group differences in average functional connectivity between the visual network and cortical nodes in isolated rapid eye movement (REM) sleep behavior disorder (iRBD) compared with controls.
**Supplementary TABLE S5.** Group differences in average functional connectivity between the basal ganglia, locus coeruleus, nucleus basalis of Meynert, and resting‐state networks in isolated rapid eye movement (REM) sleep behavior disorder (iRBD) compared with controls.
**Supplementary TABLE S6.** Cross‐sectional changes in dynamic functional connectivity in patients with isolated rapid eye movement (REM) sleep behavior disorder (iRBD) compared with healthy controls.
**Supplementary TABLE S7.** Group differences in average between and within module connectivity of resting‐state networks in isolated rapid eye movement (REM) sleep behavior disorder (iRBD) compared with controls.
**Supplementary TABLE S8.** Spearman's correlations of static and dynamic functional connectivity measures with clinical variables.
**Supplementary TABLE S9.** Longitudinal changes in static internetwork functional connectivity in patients with isolated rapid eye movement (REM) sleep behavior disorder (iRBD).
**Supplementary TABLE S10.** Longitudinal changes in subcortical functional connectivity to resting state networks in patients with isolated rapid eye movement (REM) sleep behavior disorder (iRBD).
**Supplementary TABLE S11.** Longitudinal changes in dynamic functional connectivity in patients with isolated rapid eye movement (REM) sleep behavior disorder (iRBD).
**Supplementary TABLE S12.** Longitudinal associations between cognitive performance and functional connectivity metrics in patients with isolated rapid eye movement (REM) sleep behavior disorder (iRBD).
**Supplementary TABLE S13.** Cox proportional hazards models examining the association between baseline functional connectivity measures and risk of disease conversion in isolated rapid eye movement (REM) sleep behavior disorder (iRBD) patients.
**Supplementary TABLE S14.** Longitudinal functional connectivity trajectories in converters and non‐converters.
**Supplementary TABLE S15.** Cross‐sectional Spearman's correlations between functional connectivity measures and neurotransmitter densities.
**Supplementary TABLE S16.** Cross‐sectional dominance analysis between functional connectivity measures and neurotransmitter densities.
**Supplementary TABLE S17.** Longitudinal Spearman's correlations between functional connectivity changes over time and neurotransmitter densities.
**Supplementary TABLE S18.** Longitudinal dominance analysis between functional connectivity measures and neurotransmitter densities.

## Data Availability

The data that support the findings of this study are available from the corresponding author upon reasonable request.
