# Supplementary material for: Functional Magnetic Resonance Imaging (fMRI) Signatures of Progression and Phenoconversion in Prodromal Synucleinopathies
Source: Mov Disord. 2025 Sep 1;40(12):2664–77. doi: 10.1002/mds.70025 (PMC12710112; doi:10.1002/mds.70025)
Supplement: Supplementary file 1 — Supplementary TABLE S1. Cross‐sectional and longitudinal clinical demographics in isolated rapid eye movement (REM) sleep behavior disorder (iRBD) and controls. Supplementary TABLE S2. Demographics and clinical characteristics of isolated rapid eye movement (REM) sleep behavior disorder (iRBD) patients by sex. Supplementary TABLE S3. Group differences in average functional connectivity between resting state networks and cortical nodes in isolated rapid eye movement (REM) sleep behavior disorder (iRBD) compared with controls. Supplementary TABLE S4. Group differences in average functional connectivity between the visual network and cortical nodes in isolated rapid eye movement (REM) sleep behavior disorder (iRBD) compared with controls. Supplementary TABLE S5. Group differences in average functional connectivity between the basal ganglia, locus coeruleus, nucleus basalis of Meynert, and resting‐state networks in isolated rapid eye movement (REM) sleep behavior disorder (iRBD) compared with controls. Supplementary TABLE S6. Cross‐sectional changes in dynamic functional connectivity in patients with isolated rapid eye movement (REM) sleep behavior disorder (iRBD) compared with healthy controls. Supplementary TABLE S7. Group differences in average between and within module connectivity of resting‐state networks in isolated rapid eye movement (REM) sleep behavior disorder (iRBD) compared with controls. Supplementary TABLE S8. Spearman's correlations of static and dynamic functional connectivity measures with clinical variables. Supplementary TABLE S9. Longitudinal changes in static internetwork functional connectivity in patients with isolated rapid eye movement (REM) sleep behavior disorder (iRBD). Supplementary TABLE S10. Longitudinal changes in subcortical functional connectivity to resting state networks in patients with isolated rapid eye movement (REM) sleep behavior disorder (iRBD). Supplementary TABLE S11. Longitudinal changes in dynamic functional connectivity i [file MDS-40-2664-s001.docx]

**Supplementary Materials**

**Neuropsychological tests**

Participants underwent a comprehensive neuropsychological battery including the following tests:

- **Global Cognition:**
  - Montreal Cognitive Assessment (MoCA)
- **Executive Function:**
  - Trail Making Test Part B (TMT-B)
  - Delis-Kaplan Executive Function System (D-KEFS) Color-Word Interference Test (CWIT) Condition 3: Inhibition
  - D-KEFS CWIT Condition 4: Switching and Cognitive Flexibility
- **Visuospatial Function:**
  - Clock Drawing Test
- **Attention and Working Memory:**
  - Digit Span Backward
- **Learning and Memory:**
  - Rey Auditory Verbal Learning Test (RAVLT)
  - Wechsler Memory Scale - Third Edition (WMS-III)
- **Language:**
  - Controlled Oral Word Association Test (COWAT)
- **Sensory and Motor Function:**
  - Sniffin’ Sticks olfactory test
  - Movement Disorder Society Unified Parkinson’s Disease Rating Scale Part III (MDS-UPDRS III)

**Functional pre-processing**

: For each of the 3 BOLD runs found per subject (across all tasks and sessions), the following preprocessing was performed. First, a reference volume and its skull-stripped version were generated using a custom methodology of *fMRIPrep*. Head-motion parameters with respect to the BOLD reference (transformation matrices, and six corresponding rotation and translation parameters) are estimated before any spatiotemporal filtering using mcflirt` [FSL 6.0.3:b862cdd5, @mcflirt]. BOLD runs were slice-time corrected to 1.46s (0.5 of slice acquisition range 0s-2.92s) using `3dTshift` from AFNI 20170202 [@afni, RRID:SCR_005927]. The BOLD time-series (including slice-timing correction when applied) were resampled onto their original, native space by applying the transforms to correct for head-motion. These resampled BOLD time-series will be referred to as *preprocessed BOLD in original space*, or just *preprocessed BOLD*. The BOLD reference was then co-registered to the T1w reference using `mri_coreg` (FreeSurfer) followed by `flirt` [FSL 6.0.3:b862cdd5, @flirt] with the boundary-based registration [@bbr] cost-function. Co-registration was configured with six degrees of freedom. Several confounding time-series were calculated based on the *preprocessed BOLD*: framewise displacement (FD), DVARS and three region-wise global signals. FD was computed using two formulations following Power (absolute sum of relative motions, @power_fd_dvars) and Jenkinson (relative root mean square displacement between affines, @mcflirt). FD and DVARS are calculated for each functional run, both using their implementations in *Nipype* [following the definitions by @power_fd_dvars]. The three global signals are extracted within the CSF, the WM, and the whole-brain masks. Additionally, a set of physiological regressors were extracted to allow for component-based noise correction [*CompCor*, @compcor]. Principal components are estimated after high-pass filtering the *preprocessed BOLD* time-series (using a discrete cosine filter with 128s cut-off) for the two *CompCor* variants: temporal (tCompCor) and anatomical (aCompCor). tCompCor components are then calculated from the top 2% variable voxels within the brain mask. For aCompCor, three probabilistic masks (CSF, WM and combined CSF+WM) are generated in anatomical space. The implementation differs from that of Behzadi et al. in that instead of eroding the masks by 2 pixels on BOLD space, the aCompCor masks are subtracted a mask of pixels that likely contain a volume fraction of GM. This mask is obtained by thresholding the corresponding partial volume map at 0.05, and it ensures components are not extracted from voxels containing a minimal fraction of GM. Finally, these masks are resampled into BOLD space and binarized by thresholding at 0.99 (as in the original implementation). Components are also calculated separately within the WM and CSF masks. For each CompCor decomposition, the *k* components with the largest singular values are retained, such that the retained components' time series are sufficient to explain 50 percent of variance across the nuisance mask (CSF, WM, combined, or temporal). The remaining components are dropped from consideration. The head-motion estimates calculated in the correction step were also placed within the corresponding confounds file. The confound time series derived from head motion estimates and global signals were expanded with the inclusion of temporal derivatives and quadratic terms for each [@confounds_satterthwaite_2013]. Frames that exceeded a threshold of 0.5 mm FD or 1.5 standardised DVARS were annotated as motion outliers. The BOLD time-series were resampled into several standard spaces, correspondingly generating the following *spatially-normalized, preprocessed BOLD runs*: MNI152NLin6Asym, MNI152NLin2009cAsym. First, a reference volume and its skull-stripped version were generated using a custom methodology of *fMRIPrep*. All resamplings can be performed with *a single interpolation step* by composing all the pertinent transformations (i.e. head-motion transform matrices, susceptibility distortion correction when available, and co-registrations to anatomical and output spaces). Gridded (volumetric) resamplings were performed using `antsApplyTransforms` (ANTs), configured with Lanczos interpolation to minimize the smoothing effects of other kernels [@lanczos]. Non-gridded (surface) resamplings were performed using `mri_vol2surf` (FreeSurfer).

**Denoising**

Following preprocessing with fMRIPrep, further denoising and parcellation of functional MRI data were performed using Python and the Nilearn package. Functional data in MNI152NLin6Asym space with a 2 mm resolution were parcellated using a custom atlas with 400 Schaefer Cortical regions and Tian’s Subcortical Atlases (ROIs). Denoising included confound regression to remove nuisance signals. Confounds were derived using fMRIPrep's confound extraction, incorporating motion parameters (derivatives), white matter (WM), and cerebrospinal fluid (CSF) signals. The functional time series were detrended and standardized (z-scored) before further processing. Additionally, high-pass (0.01 Hz) and low-pass (0.1 Hz) filters were applied to mitigate low-frequency drifts and high-frequency noise. Time series for each subject and session were extracted from the parcellated ROIs using the NiftiLabelsMasker function in Nilearn. For each ROI, the standardized, filtered time series were saved in MATLAB (.mat) format, with dimensions corresponding to time points by ROIs. This approach facilitated subsequent connectivity and timeseries-based analyses.

**Resting state network based static functional connectivity**

The timeseries were used to create functional connectivity matrices for each individual patient using Pearson correlation, representing regional by regional functional connectivity. The 400 cortical regions were initially assigned to the 17 resting state networks developed by Yeo et al,^63^ which were subsequently grouped into broader eight resting state networks to allow for more meaningful, large scale network comparisons. The resting state networks can be defined as the following: visual network, somatomotor network, dorsal attentional network, ventral attentional network, limbic network, frontoparietal network, default mode network and temporal network.^60,63^

Whole-cortex (global) functional connectivity was computed by averaging the pairwise functional connectivity values across all 400 cortical nodes. Network-to-cortex functional connectivity was calculated by first computing the connectivity between each node within a given network and all 400 cortical nodes, followed by averaging these values across all nodes within the network. Internetwork functional connectivity was derived by averaging the connectivity values between all node pairs belonging to two distinct networks (e.g., visual and dorsal attention networks), yielding a summary measure of connectivity strength between each network pair.

To assess the subcortical contribution to resting-state network dysfunction, we first extracted the functional connectivity matrices from the Tian subcortical parcellations corresponding to the bilateral NBM, LC, and the BG, comprised of the caudate and putamen.^59^ For each subcortical ROI, we calculated the average functional connectivity to nodes within each of the eight resting-state networks, enabling us to evaluate the overall connectivity between these structures and the resting state networks described above.

**Dynamic functional connectivity analysis**

**Temporal correlations of functional configurations**

As an initial measure of functional dynamics, we examined temporal differences in brain state configurations, categorised into two distinct metrics: Local Similarity (S_L_) and Global Similarity (S_G_). The Pearson’s correlation of BOLD activity across the whole brain computed across each time epoch (TR) of the scan.^50^

Local similarity refers to the correlation of neural activity within each parcel across consecutive timepoints during a scan. These correlations are averaged across all parcels to yield a whole-brain measure. Higher local similarity values indicate more consistent neuronal configuration patterns, reflecting a more stationary brain state over the duration of the scan. This metric provides a way to quantify the persistence of specific brain configurations over a segment of the scan.

Unlike local similarity, which focuses on consecutive time points, global similarity evaluates regional correlations across the entire scan. It is calculated as the mean correlation of configurations across all epochs in a time series, providing a measure of brain state variability throughout the scan. Higher global similarity values indicate lower variability in functional configurations.

**Temporal derivatives and community assignment**

To accurately incorporate the time-resolved community structure of functional connectivity over the scan we initially calculated the multiplication of temporal derivatives for each time series with a sliding window of 15 TRs (Code available: https://github.com/lachychurchill/coupling_integration/).^34^ Next, we implemented the Louvain Community Assignment to classify nodes into modules throughout the course of the scan and the proceeding analysis using the Brain Connectivity Toolbox^64^ (<https://sites.google.com/site/bctnet/>). Within-module connectivity was estimated by calculating the module degree Z score (W_T_) for each parcel.^65^ Higher module degree z-scores indicate that a parcel is more strongly connected with its own module, suggesting that it plays a central role in local (intramodular) processing. Between-module connectivity was calculated using the participation coefficient (B_T_) and provides insight on the extent to which a region is connected to other wide-ranging modules compared to its intramodular connectivity. Participation scores that are closer to 1 represent a wide range of intermodular connectivity compared to those that are closer to 0.

Time-resolved modularity score (Q) was also calculated in each participant, indicating the number of distinct modules the brain could be divided into at that time. A higher modularity indicates that the cortical organisation is comprised of a larger number of distinct subnetworks.^66^ The modular flexibility was also calculated by deriving the region change in modular assignment, determined by using the Hungarian algorithm, throughout the sliding windows of the scan.^67,68^ This measure describes variability of modular assignments for different regions over time.

**Characterisation of macroscopic brain states**

To capture the temporal dynamics of region-associated fluctuations in dynamic functional connectivity, we calculated B_T_ and W_T_ for each region across all sliding windows of the scan to create a relationship plot at a patient level. Based on this characterisation, we clustered brain states into two categories (*k*=2): highly integrated states (high B_T_ and low W_T_) and highly segregated states (low B_T_ and high W_T_) as previously described.^45^ These clusters provided measures of whole-brain integration and segregation across the time course of the scan for each patient. We then used these dynamic measures to analyse brain state transitions, and the characteristics associated with shifts between integrated and segregated states.

**Neurotransmitter receptor and gene receptor density mapping**

To translate the neurobiological basis into functional connectivity measures, we utilised the recently developed neurotransmitter receptor density mapping approach (Code available: <https://github.com/netneurolab/hansen_receptors>).^69^ These maps were derived from data collected from over 1,200 positron emission tomography (PET) participants, providing region-specific density estimates for cholinergic, noradrenergic, and dopaminergic neurotransmitter systems. Functional connectivity and participation coefficients for the 400 Schaefer regions were extracted and analysed statistically to assess disparities between groups. These analyses were conducted cross-sectionally (iRBD vs. controls) and longitudinally (iRBD across time points). The test statistics for each region were then correlated with the cholinergic, dopaminergic, and noradrenergic receptor densities using Spearman's rank correlation. We subsequently performed a dominance analysis to further investigate the relative contributions of each neurotransmitter system to the observed connectivity patterns.

**Statistical Analysis**

All statistical analyses were performed using MATLAB R2023b (The MathWorks, Inc., Massachusetts, United States). For the cross-sectional analysis, demographic variables were compared using chi-squared tests for binary data and independent-samples t-tests for continuous data, with 10,000 permutations conducted to account for distributional assumptions. Static and dynamic functional connectivity measures were analysed using general linear models, incorporating age, sex, and education as covariates, with disease group serving as the variable of interest. Correlations between functional connectivity measures and clinical variables were assessed within the iRBD cohort using Spearman rank correlation analysis.

For the longitudinal analysis of iRBD participants, variables were examined using a linear mixed-effects model with time (measured in months) as a continuous variable of interest with age, sex, and education as covariates. An interaction term between time and the functional connectivity measure determined how changes in functional connectivity were correlated clinical variables over time. Differences in longitudinal trajectories of converters were analysed by modelling the functional connectivity measure as the dependent variable while group and time were modelled as an interaction term. Collinearity among predictors was assessed using variance inflation factors, with all values below the commonly accepted threshold of 10, indicating no concerning multicollinearity.^70^ All statistical significance was determined using FDR correction with a threshold of *p* < 0.05.

A Cox proportional hazards model was used to assess the influence of functional connectivity measures on the relative risk of future phenoconversion. Longitudinal clinical follow-up data were available for all 41 iRBD patients initially scanned and phenotyped at baseline, of whom 10 converted to PD and 7 to DLB. Two separate survival models were examined: the first assessed conversion to any α-synucleinopathy (PD or DLB), and the second focused specifically on conversion to DLB. Follow-up time was measured in months (maximum = 126; mean = 41), with conversion defined as the event of interest and non-converters censored at their last available follow-up. To meet the recommended guideline of 5–10 events per variable, age was included as the sole covariate.^71^ Model fit was assessed using log-likelihood ratio tests, with statistical significance set at p < 0.05.

**Supplementary Tables**

**Supplementary Table 1: Cross sectional and longitudinal clinical demographics in isolated REM sleep behaviour disorder and controls**

| **Clinical Variables** | **Controls (n=38)** | **iRBD (n=41)** | **iRBD Timepoint 2 (n=21)** | **iRBD Timepoint 3 (n=6)** | **Cross sectional *p* value** | **Long *p* value** |
| --- | --- | --- | --- | --- | --- | --- |
| Age | 66.95 (7.55) | 65.93 (6.68) | 66.05 (7.74) | 67.33 (6.08) | 0.516 | - |
| Education | 14.24 (2.79) | 13.15 (3.01) | 13.43 (2.84) | 13.83 (1.72) | 0.095 | - |
| Disease Duration (years) | - | 1.94 (2.20) | 4.75 (2.66) | 5.94 (0.76) | - | - |
| Disease conversion | - | - | 28.6% (PD 3, DLB 3) | 33.3% (1 PD, 1 DLB) | - | - |
| Sex (Male : Female) | 17 : 21 | 34 : 7 | 16 : 5 | 5 : 1 | **<0.001** | - |
| Time from baseline scan (months) | - | - | 33.09 (18.87) | 56.5 (12.14) | - | - |
| Dopamine medication (% on) | - | - | 14% | 33% | - | - |
| Cholinergic medication (% on) | - | - | 4.8% | 16% | - | - |
| SSRI (% on) | 7.9% | 7.3% | 0% | 0% | 0.999 | - |
| SNRI (% on) | 2.6% | 0% | 0% | 0% | 0.969 | - |
| Benzodiazepines (% on) | 5.3% | 9.8% | 14% | 16% | 0.742 | - |
| **Questionnaires** | | | | | | |
| SCOPA Sleep Night | 4.06 (3.62) | 2.66 (3.25) | 3.50 (2.56) | 4.5 (2.66) | 0.148 | 0.526 |
| SCOPA Sleep Day | 2.28 (2.11) | 2.66 (3.64) | 3 (2.77) | 4.33 (4.63) | 0.692 | 0.525 |
| Epworth Sleepiness Scale | 5.23 (3.20) | 7.06 (5.54) | 6.1 (4.52) | 8.16 (6.82) | 0.148 | 0.741 |
| RBD Screening Questionnaire | 2.59 (2.04) | 7.52 (3.23) | 7.75 (3.39) | 10.16 (1.47) | **<0.001** | 0.665 |
| HADS Anxiety | 2.64 (2.16) | 3.13 (3.41) | 3.65 (3.36) | 5.83 (4.83) | 0.648 | 0.264 |
| HADS Depression | 1.76 (1.81) | 2.55 (3.44) | 2.95 (3.24) | 3.16 (2.56) | 0.704 | 0.585 |
| MDS-UPDRS Question 1.2 (Visual Hallucination Score) | 0 (0) | 0.06 (0.24) | 0.26 (0.56) | 1.67 (0.41) | 0.144 |  |
| **Cognitive Measures** | | | | | | |
| Montreal Cognitive Assessment | 28.03 (2.04) | 26.68 (2.58) | 25.65 (3.73) | 27.5 (3.674) | **0.012** | 0.133 |
| Trail Making B | 0.59 (0.63) | 0.12 (1.10) | 0.26 (1.05) | 0.47 (0.58) | **0.037** | 0.986 |
| Digit Span Backwards | 12.50 (2.76) | 11.77 (2.94) | 12.05 (3.38) | 12.00 (4.04) | 0.327 | 0.856 |
| Stroop Inhibition | 11.68 (3.09) | 11.11 (2.6) | 10.95 (3.3) | 12.00 (0.71) | 0.467 | 0.479 |
| Stroop Inhibition/Switching | 12.18 (2.48) | 11.31 (2.8) | 10.85 (3.07) | 11.00 (1.58) | 0.248 | **0.057** |
| **Sensory/Motor** | | | | | | |
| Sniffin Sticks | 10.13 (1.36) | 7.10 (2.96) | 6.42 (2.81) | 5.16 (1.60) | **<0.001** | 0.991 |
| UPDRS III | 1.26 (1.81) | 9.17 (8.32) | 11.95 (9.98) | 6.33 (5.64) | **<0.001** | 0.428 |

Values are displayed as mean (standard deviation). Participants were matched on Age and Education but differed significantly on Sex. P values were calculated using non-parametric permutation testing and chi-squared tests where applicable. Longitudinal p values were calculated using a linear mixed effects model using time in months. DLB, Dementia with Lewy Bodies; HADS, Hospital Anxiety and Depression Scale; PD, Parkinson’s Disease; SCOPA, Scales for Outcomes in Parkinson’s disease; UPDRS III, Unified Parkinson’s Disease Rating Scale Section III.

**Supplementary Table 2: Demographics and clinical characteristics of idiopathic REM sleep behaviour patients by sex**

| **Clinical Variables** | **Male iRBD (*n*= 34)** | **Female iRBD (*n*=7)** | |
| --- | --- | --- | --- |
| Age | 66.47 (6.91) | 63.29 (4.99) | |
| Education | 13.38 (2.75) | 12.00 (4.12) | |
| Disease Duration (years) | 2.10 (2.26) | 1.45 (0.34) | |
| SSRI (% on) | 5.9% | 14.2% | |
| SNRI (% on) | 0% | 0% | |
| Benzodiazepines (% on) | 8.8% | 14.2% | |
| **Questionnaires** | | |  |
| SCOPA Sleep Night | 2.29 (3.27) | 4.29 (2.81) | |
| SCOPA Sleep Day | 2.52 (3.57) | 3.29 (4.19) | |
| Epworth Sleepiness Scale | 7.04 (5.72) | 7.20 (4.97) | |
| RBD Screening Questionnaire | 7.63 (3.05) | 7.00 (4.24) | |
| HADS Anxiety | 3.03 (3.25) | 3.57 (4.31) | |
| HADS Depression | 2.68 (3.49) | 2.00 (3.42) | |
| **Cognitive Measures** | | |  |
| Montreal Cognitive Assessment | 26.68 (2.47) | 26.57 (3.26) | |
| Trail Making Test B | 0.32 (0.84) | -0.69 (1.69) | |
| Digit Span Backwards | 11.93 (3.05) | 11.00 (2.45) | |
| Stroop Inhibition | 11.21 (2.29) | 10.67 (4.08) | |
| Stroop Inhibition/Switching | 11.34 (2.77) | 11.17 (3.31) | |
| **Sensory/Motor** | | |  |
| Sniffin Sticks | 6.91 (3.15) | 7.83 (2.23) | |
| MDS-UPDRS III | 9.67 (8.56) | 6.20 (6.69) | |

Values are displayed as mean (standard deviation). P values were calculated using non-parametric permutation testing and chi-squared tests where applicable. DLB, Dementia with Lewy Bodies; HADS, Hospital Anxiety and Depression Scale; MDS-UPDRS III, Movement disorder society unified Parkinson’s disease rating scale; PD, Parkinson’s Disease; SCOPA, Scales for Outcomes in Parkinson’s disease; UPDRS III, Unified Parkinson’s Disease Rating Scale Section III.

**Supplementary Table 3: Group differences in average functional connectivity between resting state networks and cortical nodes in iRBD compared to controls**

| **Regions** | **VIS** | **SMN** | **DAN** | **VAN** | **LIM** | **FPN** | **DMN** | **TEM** | **Whole cortex** |
| --- | --- | --- | --- | --- | --- | --- | --- | --- | --- |
| observed T statistic | **-2.70** | -1.27 | -1.52 | -0.62 | -1.14 | -0.82 | -1.75 | -1.90 | -1.93 |
| P value | **0.008** | 0.226 | 0.130 | 0.532 | 0.231 | 0.406 | 0.097 | 0.056 | 0.099 |
| FDR corrected (p<0.05) | 0.060 | 0.308 | 0.260 | 0.532 | 0.308 | 0.464 | 0.259 | 0.224 | 0.124 |
| Cohen’s d | -0.68 | -0.33 | -0.39 | -0.16 | -0.29 | -0.21 | -0.45 | -0.49 | -0.42 |

T statistic was calculated using a general linear model with Age, Sex and Education as covariates. DAN, Dorsal attentional network; DMN, Default mode network; FC, Functional connectivity; FPN, Fronto-parietal network; LIM, Limbic network; SMN, Somatomotor network; TEM, Temporal network; VAN, Ventral attentional network; VIS, Visual network

**Supplementary Table 4: Group differences in average functional connectivity between the visual network and cortical nodes in iRBD compared to controls.**

| **Regions** | **VIS - VIS** | **VIS - SMN** | **VIS - DAN** | **VIS - VAN** | **VIS - LIM** | **VIS - FPN** | **VIS - DMN** | **VIS - TEM** |
| --- | --- | --- | --- | --- | --- | --- | --- | --- |
| observed T statistic | **-3.13** | -2.34 | -2.11 | -0.85 | -1.74 | -0.93 | **-2.69** | -2.18 |
| 2 sided P value | **0.003** | 0.024 | 0.037 | 0.393 | 0.080 | 0.356 | **0.005** | 0.039 |
| FDR corrected (p<0.05) | **0.020** | 0.062 | 0.062 | 0.393 | 0.106 | 0.393 | **0.020** | 0.062 |
| Cohen’s d | **-0.82** | -0.60 | -0.54 | -0.22 | -0.45 | -0.24 | **-0.69** | -0.56 |

T statistic was calculated using a general linear model with Age, Sex and Education as covariates. DAN, Dorsal attentional network; DMN, Default mode network; FC, Functional connectivity; FPN, Fronto-parietal network; LIM, Limbic network; SMN, Somatomotor network; TEM, Temporal network; VAN, Ventral attentional network; VIS, Visual network

**Supplementary Table 5: Group differences in average functional connectivity between the basal ganglia, locus coeruleus, nucleus basalis of Meynert, and resting-state networks in iRBD compared to controls.**

| **Basal ganglia** | **VIS** | **SMN** | **DAN** | **VAN** | **LIM** | **FPN** | **DMN** | **TEM** |
| --- | --- | --- | --- | --- | --- | --- | --- | --- |
| observed T statistic | -0.46 | 0.41 | 0.05 | -0.39 | -0.85 | -0.50 | -1.62 | -0.88 |
| 2-sided P value | 0.648 | 0.685 | 0.955 | 0.689 | 0.397 | 0.619 | 0.107 | 0.378 |
| **Nucleus basalis of Meynert** | **VIS** | **SMN** | **DAN** | **VAN** | **LIM** | **FPN** | **DMN** | **TEM** |
| observed T statistic | 0.99 | 0.26 | 0.65 | -1.83 | 1.14 | -0.45 | 1.16 | -0.43 |
| 2-sided P value | 0.314 | 0.800 | 0.517 | 0.071 | 0.257 | 0.660 | 0.255 | 0.671 |
| **Locus coruleus** | **VIS** | **SMN** | **DAN** | **VAN** | **LIM** | **FPN** | **DMN** | **TEM** |
| observed T statistic | -1.24 | 0.77 | -1.25 | -0.12 | -1.44 | -1.36 | -1.28 | 0.31 |
| 2-sided P value | 0.219 | 0.443 | 0.222 | 0.905 | 0.154 | 0.175 | 0.202 | 0.765 |

T statistic was calculated using a general linear model with Age, Sex and Education as covariates. DAN, Dorsal attentional network; DMN, Default mode network; FC, Functional connectivity; FPN, Fronto-parietal network; LIM, Limbic network; SMN, Somatomotor network; TEM, Temporal network; VAN, Ventral attentional network; VIS, Visual network

**Supplementary Table 6: Cross-sectional changes in dynamic functional connectivity in patients with isolated REM sleep behviour disorder compared to healthy controls.**

|  | **Raw Value Control** | **Raw Value iRBD** | **T statistic** | ***p* value** | **Cohen’s d** |
| --- | --- | --- | --- | --- | --- |
| **Modularity** | 0.33 (0.05) | 0.35 (0.05) | 2.12 | **0.039** | **0.55** |
| **Local Similarity** | 0.54 (0.04) | 0.52 (0.04) | -2.03 | **0.042** | **-0.52** |
| **Global Similarity** | 0.17 (0.02) | 0.17 (0.02) | 0.04 | 0.973 | -0.01 |
| **Participation Coefficient (B_T_)** | 0.45 (0.04) | 0.45 (0.04) | -0.94 | 0.352 | -0.24 |
| **Module Degree Z-Score (W_T_)** | 0.01 (0.16) | 0.01 (0.19) | 0.21 | 0.838 | 0.05 |
| **Dwell Time** | 7.41 (2.75) | 7.25 (2.73) | -0.29 | 0.777 | -0.10 |

Values represent the observed raw values, T statistic, two-sided p-value and cohen’s d for whole brain dynamic functional connectivity measures. Calculations were performed using a general linear model, with age, sex, and education included as covariates. B_T_, Participation coefficient; S_L_, Local similarity, S_G_, Global similarity; Q, Modularity; W_T_, Module degree-score

**Supplementary Table 7: Group differences in average between and within module connectivity of resting-state networks in iRBD compared to controls.**

| **Participation (B_T_)** | **VIS** | **SMN** | **DAN** | **VAN** | **LIM** | **FPN** | **DMN** | **TEM** |
| --- | --- | --- | --- | --- | --- | --- | --- | --- |
| observed T statistic | -1.32 | -0.92 | -0.822 | -0.69 | -0.42 | -0.60 | -1.22 | -0.82 |
| 2-sided P value | 0.191 | 0.354 | 0.416 | 0.481 | 0.672 | 0.542 | 0.227 | 0.415 |
| **Module Degree Z-Score (W_T_)** | **VIS** | **SMN** | **DAN** | **VAN** | **LIM** | **FPN** | **DMN** | **TEM** |
| observed T statistic | -1.87 | 1.039 | -0.099 | 1.415 | -0.64 | 0.32 | -0.908 | -1.73 |
| 2-sided P value | 0.062 | 0.301 | 0.927 | 0.158 | 0.524 | 0.75 | 0.360 | 0.089 |

T statistic was calculated using a general linear model with Age, Sex and Education as covariates. DAN, Dorsal attentional network; DMN, Default mode network; FC, Functional connectivity; FPN, Fronto-parietal network; LIM, Limbic network; SMN, Somatomotor network; TEM, Temporal network; VAN, Ventral attentional network; VIS, Visual network

**Supplementary Table 8: Spearman’s correlations of static and dynamic functional connectivity measures with clinical variables.**

| **MoCA** | **VIS WB** | **VIS VIS** | **VIS DMN** | **Q** | **B_T_** | **W_T_** | **S_L_** | **S_G_** | **Dwell Time** | **Transition** |
| --- | --- | --- | --- | --- | --- | --- | --- | --- | --- | --- |
| observed R statistic | 0.142 | 0.219 | 0.097 | -0.035 | -0.002 | 0.259 | 0.246 | 0.062 | -0.103 | 0.108 |
| 2-sided P value | 0.396 | 0.186 | 0.564 | 0.834 | 0.988 | 0.116 | 0.136 | 0.712 | 0.540 | 0.519 |
| FDR correction (p<0.05) | 0.820 | 0.620 | 0.820 | 0.920 | 0.980 | 0.620 | 0.620 | 0.880 | 0.820 | 0.820 |
| **TMT-B** | **VIS WB** | **VIS VIS** | **VIS DMN** | **Q** | **B_T_** | **W_T_** | **S_L_** | **S_G_** | **Dwell Time** | **Transition** |
| observed R statistic | -0.004 | 0.086 | -0.094 | -0.150 | 0.154 | 0.298 | **0.358** | -0.033 | 0.265 | **-0.427** |
| 2-sided P value | 0.983 | 0.612 | 0.580 | 0.374 | 0.363 | 0.073 | **0.030** | 0.844 | 0.112 | **0.008** |
| FDR correction (p<0.05) | 0.980 | 0.760 | 0.760 | 0.620 | 0.620 | 0.240 | 0.150 | 0.930 | 0.280 | 0.080 |
| **Digit Span** | **VIS WB** | **VIS VIS** | **VIS DMN** | **Q** | **B_T_** | **W_T_** | **S_L_** | **S_G_** | **Dwell Time** | **Transition** |
| observed R statistic | 0.080 | -0.039 | 0.179 | 0.043 | 0.079 | 0.210 | -0.177 | -0.102 | -0.156 | 0.027 |
| 2-sided P value | 0.647 | 0.825 | 0.304 | 0.805 | 0.651 | 0.226 | 0.310 | 0.560 | 0.370 | 0.878 |
| FDR correction (p<0.05) | 0.878 | 0.878 | 0.878 | 0.878 | 0.878 | 0.878 | 0.878 | 0.878 | 0.878 | 0.878 |
| **MDS-UPDRS III** | **VIS WB** | **VIS VIS** | **VIS DMN** | **Q** | **B_T_** | **W_T_** | **S_L_** | **S_G_** | **Dwell Time** | **Transition** |
| observed R statistic | 0.039 | 0.039 | 0.010 | -0.126 | 0.128 | -0.364 | -0.023 | -0.089 | -0.098 | 0.001 |
| 2-sided P value | 0.823 | 0.825 | 0.955 | 0.471 | 0.463 | 0.032 | 0.894 | 0.610 | 0.577 | 0.997 |
| FDR correction (p<0.05) | 0.997 | 0.997 | 0.997 | 0.997 | 0.997 | 0.320 | 0.997 | 0.997 | 0.997 | 0.997 |

Spearmans correlation calculated between clinical variables and corresponding static and dynamic functional connectivity variables. B_T_, Between module connectivity; DMN, Default mode network; FC, Functional connectivity; FPN, Fronto-parietal network; LIM, Limbic network; MDS-UPDRS III, Movement disorders society unified Parkinson’s disease rating scale section III; MoCA, Montreal cognitive assessment; Q, Modularity, S_L_, Local similarity; S_G_, Global similarity; SMN, Somatomotor network; TMT-B, Trail making test B; VIS, Visual network; W_T_, Within module connectivity; WB, Whole brain.

**Supplementary Table 9: Longitudinal changes in static internetwork functional connectivity in patients with isolated REM sleep behviour disorder.**

| **Networks to entire cortex** | **VIS** | **SMN** | **DAN** | **VAN** | **LIM** | **FPN** | **DMN** | **TEM** |
| --- | --- | --- | --- | --- | --- | --- | --- | --- |
| observed T statistic | -1.85 | -3.56 | -2.63 | -2.94 | -2.07 | -1.32 | -1.00 | -1.88 |
| 2-sided P value | 0.069 | **<0.001** | **0.010** | **0.004** | 0.042 | 0.188 | 0.325 | 0.064 |
| FDR correction (p<0.05) | 0.092 | **0.005** | **0.028** | **0.017** | 0.084 | 0.214 | 0.325 | 0.092 |
| **SMN to networks** | **VIS** | **SMN** | **DAN** | **VAN** | **LIM** | **FPN** | **DMN** | **TEM** |
| observed T statistic | -2.26 | -5.16 | -3.55 | -3.90 | -3.27 | -0.79 | -0.50 | -2.42 |
| 2-sided P value | **0.027** | **<0.001** | **0.001** | **<0.001** | **0.002** | 0.435 | 0.622 | **0.018** |
| FDR correction (p<0.05) | **0.036** | **<0.001** | **0.002** | **0.001** | **0.003** | 0.497 | 0.622 | **0.029** |
| **DAN to networks** | **VIS** | **SMN** | **DAN** | **VAN** | **LIM** | **FPN** | **DMN** | **TEM** |
| observed T statistic | -1.99 | -3.55 | -2.90 | -2.64 | -1.69 | -0.83 | -0.71 | -2.07 |
| 2-sided P value | 0.051 | **0.001** | **0.005** | **0.011** | 0.095 | 0.408 | 0.482 | 0.042 |
| FDR correction (p<0.05) | 0.082 | **0.006** | **0.021** | **0.028** | 0.126 | 0.466 | 0.482 | 0.082 |
| **VAN to networks** | **VIS** | **SMN** | **DAN** | **VAN** | **LIM** | **FPN** | **DMN** | **TEM** |
| observed T statistic | -0.36 | -3.90 | -2.64 | -3.14 | -3.09 | -1.87 | -0.92 | -1.91 |
| 2-sided P value | 0.722 | **<0.001** | **0.011** | **0.003** | **0.003** | 0.067 | 0.363 | 0.060 |
| FDR correction (p<0.05) | 0.722 | **0.002** | **0.021** | **0.008** | **0.008** | 0.208 | 0.524 | 0.125 |

Values represent the observed T statistic, uncorrected two-sided p-value, and FDR-corrected p-value for functional connectivity between resting-state networks and the whole cortex or other networks. Calculations were performed using a linear mixed-effects model, with time as the variable of interest and age, sex, and education included as covariates. DAN, Dorsal attentional network; DMN, Default mode network; FDR. False discovery rate; FC, Functional connectivity; FPN, Fronto-parietal network; LIM, Limbic network; SMN, Somatomotor network; TEM, Temporal network; VAN, Ventral attentional network; VIS, Visual network.

**Supplementary Table 10: Longitudinal changes in subcortical functional connectivity to resting state networks in patients with isolated REM sleep behviour disorder.**

| **Basal ganglia** | **VIS** | **SMN** | **DAN** | **VAN** | **LIM** | **FPN** | **DMN** | **TEM** |
| --- | --- | --- | --- | --- | --- | --- | --- | --- |
| observed T statistic | 1.63 | -0.99 | -0.41 | -2.38 | 1.12 | -0.81 | -0.49 | -2.02 |
| 2-sided P value | 0.871 | 0.325 | 0.685 | 0.020 | 0.267 | 0.422 | 0.624 | 0.048 |
| FDR correction (p<0.05) | 0.871 | 0.650 | 0.783 | 0.160 | 0.65 | 0.675 | 0.783 | 0.192 |
| **Nucleus basalis of Meynert** | **VIS** | **SMN** | **DAN** | **VAN** | **LIM** | **FPN** | **DMN** | **TEM** |
| observed T statistic | 0.81 | -1.19 | -0.19 | -0.04 | 0.36 | 0.09 | -0.71 | 0.29 |
| 2-sided P value | 0.422 | 0.236 | 0.850 | 0.967 | 0.722 | 0.931 | 0.477 | 0.766 |
| FDR correction (p<0.05) | 0.967 | 0.967 | 0.967 | 0.967 | 0.967 | 0.967 | 0.967 | 0.967 |
| **Locus coeruleus** | **VIS** | **SMN** | **DAN** | **VAN** | **LIM** | **FPN** | **DMN** | **TEM** |
| observed T statistic | 1.19 | 0.63 | 0.58 | 0.96 | 0.19 | 2.39 | 0.91 | -0.31 |
| 2-sided P value | 0.238 | 0.530 | 0.564 | 0.343 | 0.848 | 0.019 | 0.366 | 0.757 |
| FDR correction (p<0.05) | 0.732 | 0.752 | 0.752 | 0.732 | 0.848 | 0.152 | 0.732 | 0.848 |

Values represent the observed T statistic, uncorrected two-sided p-value, and FDR-corrected p-value for functional connectivity between the basal ganglia, nucleus basalis of Meynert, and locus coeruleus and resting-state networks. Calculations were performed using a linear mixed-effects model, with time as the variable of interest and age, sex, and education included as covariates. DAN, Dorsal attentional network; DMN, Default mode network; FDR. False discovery rate; FC, Functional connectivity; FPN, Fronto-parietal network; LIM, Limbic network; SMN, Somatomotor network; TEM, Temporal network; VAN, Ventral attentional network; VIS, Visual network

**Supplementary Table 11: Longitudinal changes in dynamic functional connectivity in patients with isolated REM sleep behviour disorder.**

| **Whole brain dynamic measures** | **Q** | **S_L_** | **S_G_** | **B_T_** | **W_T_** | **Dwell Time** | **Transitions** | **Flexibility** |
| --- | --- | --- | --- | --- | --- | --- | --- | --- |
| observed T statistic | **2.33** | 1.18 | 0.08 | **-2.00** | -1.18 | 0.74 | -0.65 | -0.21 |
| 2-sided P value | **0.023** | 0.242 | 0.942 | **0.049** | 0.241 | 0.463 | 0.517 | 0.827 |
| **Network participation (B_T_)** | **VIS** | **SMN** | **DAN** | **VAN** | **LIM** | **FPN** | **DMN** | **TEM** |
| observed T statistic | -0.95 | -1.86 | **-2.46** | **-2.24** | **-2.21** | -1.66 | -1.88 | **-2.33** |
| 2-sided P value | 0.346 | 0.067 | **0.017** | **0.028** | **0.030** | 0.101 | 0.065 | **0.023** |
| **Network module degree z-score** | **VIS** | **SMN** | **DAN** | **VAN** | **LIM** | **FPN** | **DMN** | **TEM** |
| observed T statistic | -1.88 | **-2.85** | 0.17 | -0.62 | 0.65 | **3.41** | 0.92 | 0.33 |
| 2-sided P value | 0.065 | **0.006** | 0.868 | 0.534 | 0.516 | **0.001** | 0.362 | 0.744 |

Values represent the observed T statistic and two-sided p-value for whole brain dynamic functional connectivity measures, and network based participation and module degree z-score. Calculations were performed using a linear mixed-effects model, with time as the variable of interest and age, sex, and education included as covariates. B_T_, Participation coefficient; DAN, Dorsal attentional network; DMN, Default mode network; FDR. False discovery rate; FC, Functional connectivity; FPN, Fronto-parietal network; LIM, Limbic network; S_L_, Local similarity, S_G_, Global similarity; Q, Modularity; SMN, Somatomotor network; TEM, Temporal network; VAN, Ventral attentional network; VIS, Visual network; W_T_, Module degree-score

**Supplementary Table 12: Longitudinal associations between cognitive performance and functional connectivity metrics in patients with isolated REM sleep behaviour disorder.**

| **MoCA** | **SMN WB** | **DAN WB** | **VAN WB** | **B_T_** | **S_L_** | **S_G_** | **Dwell Time** | **Transitions** |
| --- | --- | --- | --- | --- | --- | --- | --- | --- |
| observed T statistic | 1.51 | 1.18 | 1.03 | -0.26 | -1.08 | -0.46 | **-4.85** | 1.59 |
| 2-sided P value | 0.132 | 0.24435 | 0.3051 | 0.794 | 0.284 | 0.645 | **<0.001** | 0.117 |
| FDR correction (p<0.05) | 0.34 | 0.39 | 0.39 | 0.79 | 0.39 | 0.73 | **<0.001** | 0.34 |
| **TMT-B** | **SMN WB** | **DAN WB** | **VAN WB** | **B_T_** | **S_L_** | **S_G_** | **Dwell Time** | **Transitions** |
| observed T statistic | **4.461** | **3.026** | **5.44** | **-2.15** | **-4.55** | **2.96** | -1.40 | **4.188** |
| 2-sided P value | **<0.001** | **0.003** | **<0.001** | **0.035** | **<0.001** | **0.004** | 0.166 | **<0.001** |
| FDR correction (p<0.05) | **<0.001** | **0.005** | **<0.001** | **0.040** | **<0.001** | **0.005** | 0.160 | **<0.001** |
| **Digit span** | **SMN WB** | **DAN WB** | **VAN WB** | **B_T_** | **S_L_** | **S_G_** | **Dwell Time** | **Transitions** |
| observed T statistic | -1.34 | -1.37 | -1.66 | -0.64 | 0.58 | 0.099 | **2.91** | -0.87 |
| 2-sided P value | 0.187 | 0.1767 | 0.102 | 0.525 | 0.563 | 0.921 | **0.0052** | 0.386 |
| FDR correction (p<0.05) | 0.370 | 0.370 | 0.370 | 0.640 | 0.640 | 0.921 | **0.041** | 0.610 |
| **MDS-UPDRS III** | **SMN WB** | **DAN WB** | **VAN WB** | **B_T_** | **S_L_** | **S_G_** | **Dwell Time** | **Transitions** |
| observed T statistic | -0.21 | -1.03 | -0.08 | 0.69 | -0.01 | -0.13 | 0.25 | -0.61 |
| 2-sided P value | 0.836 | 0.307 | 0.939 | 0.489 | 0.998 | 0.895 | 0.799 | 0.544 |
| FDR correction (p<0.05) | 0.998 | 0.998 | 0.998 | 0.998 | 0.998 | 0.998 | 0.998 | 0.998 |

Values represent the observed T statistic, uncorrected two-sided p-value, and FDR-corrected p-value for the association between clinical variables and functional connectivity measures over time. Linear mixed-effects models were used, with time × connectivity as the interaction term of interest, and age, sex, and education included as covariates. B_T_, Participation coefficient; DAN, Dorsal attentional network; FDR. False discovery rate; MDS-UPDRS III, Movement disorders society unified Parkinson’s disease rating scale section III; S_L_, Local similarity, S_G_, Global similarity; Q, Modularity; SMN, Somatomotor network; VAN, Ventral attentional network; W_T_, Module degree-score

**Table 13: Cox proportional hazards models examining the association between baseline functional connectivity measures and risk of disease conversion in isolated REM sleep behaviour disorder patients.**

| **Clinical variables** | | |
| --- | --- | --- |
| **MoCA** | **Conversion** | **Conversion to DLB** |
| Hazard Ratio (95% confidence interval) | **1.44 (1.14 - 1.81)** | **1.76 (1.18 - 2.63)** |
| *p* value | **0.002** | **0.006** |
| Log-likelihood ratio tests | **0.001** | **<0.001** |
| **Trail making test B** | **Conversion** | **Conversion to DLB** |
| Hazard Ratio (95% confidence interval) | **2.00 (1.12 - 3.56)** | **2.98 (1.13 - 7.82)** |
| *p* value | **0.019** | **0.026** |
| Log-likelihood ratio tests | **0.024** | **0.013** |
| **MDS-UPDRS III** | **Conversion** | **Conversion to DLB** |
| Hazard Ratio (95% confidence interval) | 0.98 (0.92 - 1.05) | 0.95 (0.85 - 1.06) |
| *p* value | 0.57 | 0.32 |
| Log-likelihood ratio tests | 0.15 | 0.096 |
| **Static functional connectivity measures** | | |
| **Average VIS FC** | **Conversion** | **Conversion to DLB** |
| Hazard Ratio (95% confidence interval) | 0.94 (0.57 - 1.55) | 0.71 (0.33 - 1.55) |
| *p* value | 0.80 | 0.40 |
| Log-likelihood ratio tests | 0.35 | 0.066 |
| **Locus coeruleus - VIS** | **Conversion** | **Conversion to DLB** |
| Hazard Ratio (95% confidence interval) | **1.66 (1.00 - 2.76)** | **2.26 (1.13 - 6.16)** |
| *p* value | **0.048** | **0.025** |
| Log-likelihood ratio tests | **0.050** | **0.006** |
| **Nucleus basalis of Meynert - VIS** | **Conversion** | **Conversion to DLB** |
| Hazard Ratio (95% confidence interval) | 0.90 (0.53 - 1.50) | 1.006 (0.44 - 2.31) |
| *p* value | 0.68 | 0.98 |
| Log-likelihood ratio tests | 0.34 | 0.096 |
| **Basal ganglia - VIS** | **Conversion** | **Conversion to DLB** |
| Hazard Ratio (95% confidence interval) | 0.77 (0.48 - 1.25) | 0.88 (0.43 - 1.82) |
| *p* value | 0.29 | 0.73 |
| Log-likelihood ratio tests | 0.21 | 0.09 |
| **Dynamic functional connectivity measures** | | |
| **S_L_** | **Conversion** | **Conversion to DLB** |
| Hazard Ratio (95% confidence interval) | 1.11 (0.65 - 1.88) | 1.54 (0.61 - 3.86) |
| *p* value | 0.72 | 0.36 |
| Log-likelihood ratio tests | 0.35 | 0.060 |
| **S_G_** | **Conversion** | **Conversion to DLB** |
| Hazard Ratio (95% confidence interval) | 1.07 (0.60 - 1.94) | 1.14 (0.46 - 2.84) |
| *p* value | 0.81 | 0.78 |
| Log-likelihood ratio tests | 0.36 | 0.092 |
| **Q** | **Conversion** | **Conversion to DLB** |
| Hazard Ratio (95% confidence interval) | 0.88 (0.54 - 1.42) | 1.59 (0.68 - 3.74) |
| *p* value | 0.59 | 0.28 |
| Log-likelihood ratio tests | 0.32 | 0.052 |
| **B_T_** | **Conversion** | **Conversion to DLB** |
| Hazard Ratio (95% confidence interval) | 1.25 (0.79 - 1.98) | 0.64 (0.23 - 1.80) |
| *p* value | 0.34 | 0.39 |
| Log-likelihood ratio tests | 0.24 | 0.063 |

Cox proportional hazards models were used to assess the risk of conversion to either Parkinson’s disease (PD) or dementia with Lewy bodies (DLB), or specifically to DLB, with age included as a covariate. Functional connectivity values (static and dynamic) were z-scored to enhance interpretability of hazard ratios and 95% confidence intervals. Hazard ratios reflect the relative risk of conversion per unit decrease in Montreal Cognitive Assessment and Movement Disorder Society-Unified Parkinson’s Disease Rating Scale Part III scores, and per one-standard deviation decrease in Trail Making Test Part B and functional connectivity measures. B_T_, Participation coefficient; DLB, Dementia with Lewy bodies; FC, Functional connectivity; MoCA, Montreal Cognitive Assessment; MDS-UPDRS III, Movement Disorder Society-Unified Parkinson’s Rating Scale Section III; Q, Modularity; S_L_, Local similarity; S_G_, Global similarity; VIS, Visual network.

**Table 14: Longitudinal functional connectivity trajectories in converters and non-converters**

| **Phenoconversion to either Parkinson’s disease or dementia with Lewy bodies** | | |  |
| --- | --- | --- | --- |
| **Functional Connectivity Measure** | **Observed T statistic** | ***p* value** | |
| **Average cortical FC** | -1.00 | 0.320 | |
| **Average SMN FC** | -0.55 | 0.581 | |
| **Average VAN FC** | -0.97 | 0.331 | |
| **Average DAN FC** | -0.77 | 0.443 | |
| **Q** | 0.08 | 0.936 | |
| **B_T_** | 0.52 | 0.600 | |
| **W_T_** | **-2.28** | **0.026** | |
| **S_L_** | 0.39 | 0.701 | |
| **S_G_** | -1.01 | 0.314 | |
| **Phenoconversion to dementia with Lewy bodies** | | | |
| **Functional Connectivity Measure** | **Observed T statistic** | ***p* value** | |
| **Average cortical FC** | **-2.67** | **0.010** | |
| **Average SMN FC** | **-2.32** | **0.023** | |
| **Average VAN FC** | **-2.01** | **0.048** | |
| **Average DAN FC** | **-3.30** | **0.001** | |
| **Q** | 1.55 | 0.124 | |
| **B_T_** | -0.52 | 0.605 | |
| **W_T_** | 1.29 | 0.200 | |
| **S_L_** | 1.37 | 0.176 | |
| **S_G_** | -0.39 | 0.698 | |

Linear mixed effects models were used to examine predictors of phenoconversion to either Parkinson’s disease (PD) or Dementia with Lewy bodies (DLB), and specifically to DLB, with Age, Sex, and Education as covariates, and time and phenoconversion as an interaction term. The observed T-statistics and p-values for the interaction term (time*converter) highlight differences in functional connectivity trajectories between patients who converted to PD or DLB and those who did not. Significant results indicate differences in how specific functional connectivity measures changed over time between converters and non-converters. DAN, Dorsal attentional network; FC, Functional connectivity; Q, Modularity; S_L_, Local similarity; S_G_, Global similarity; SMN, Somatomotor network; VAN, Ventral attentional network; W_T_, Module degree z-score.

**Supplementary Table 15: Cross sectional spearmans correlations between functional connectivity measures and neurotransmitter densities.**

| **Neurotransmitter correlations with static functional connectivity** | | | | | | | | |
| --- | --- | --- | --- | --- | --- | --- | --- | --- |
| **Functional Connectivity** | **A_4_B_2_** | **M1** | **VAChT** | **D1** | **D2** | **DAT** | **NET** |  |
| observed R statistic | **-0.224** | -0.049 | **-0.223** | -0.082 | **-0.164** | -0.094 | -0.067 |  |
| 2-sided P value | **<0.001** | 0.33262 | **<0.001** | 0.09 | **<0.001** | 0.059 | 0.18 |  |
| **Neurotransmitter correlations with dynamic between module connectivity** | | | | | | | |  |
| **Participation (B_T_)** | **A_4_B_2_** | **M1** | **VAChT** | **D1** | **D2** | **DAT** | **NET** |  |
| observed R statistic | -0.04 | 0.02 | **-0.113** | -0.093 | -0.027 | -0.066 | -0.0245 |  |
| 2-sided P value | 0.422 | 0.69 | **0.023** | 0.061 | 0.594 | 0.185 | 0.623 |  |

Values represent the observed Spearman’s R statistic and two-sided p-value for the correlation between neurotransmitter/gene expression density maps and functional connectivity metrics across 400 cortical regions.
Functional connectivity values were estimated using general linear models controlling for age, sex, and education. Analyses were performed for both average functional connectivity strength and participation coefficient (B_T_). A_4_B_2_, Alpha-4 Beta-2 Nicotinic Acetylcholine Receptor; B_T_, Participation coefficient; D1, Dopamine D1 Receptor; D2, Dopamine D2 Receptor; DAT, Dopamine Transporter; M1, Muscarinic Acetylcholine Receptor M1; NET, Norepinephrine Transporter; VAChT, Vesicular Acetylcholine Transporter

**Supplementary Table 16. Cross sectional dominance analysis between functional connectivity measures and neurotransmitter densities.**

| **Neurotransmitter dominance with static functional connectivity** | | | | | | | | |
| --- | --- | --- | --- | --- | --- | --- | --- | --- |
| **Functional Connectivity** | **A4B2** | **VAChT** | **D2** | **NET** | **D1** | **DAT** | **M1** |  |
| Interactional Dominance | 0.041705 | 0.027568 | 0.0114 | 0.016742 | 0.008711 | 0.000992 | 0.000072 |  |
| Individual Dominance | 0.048469 | 0.050584 | 0.024191 | 0.006 | 0.005717 | 0.009475 | 0.00196 |  |
| Average Partial Dominance | 0.038562 | 0.032943 | 0.017294 | 0.0083 | 0.005089 | 0.004675 | 0.00267 |  |
| Total Dominance | 0.040426 | 0.034695 | 0.017438 | 0.00918 | 0.00569 | 0.00483 | 0.00219 |  |
| Percentage Relative Importance | 35.316212 | 30.309897 | 15.233751 | 8.02057 | 4.976218 | 4.2233 | 1.91995 |  |
| **Neurotransmitter dominance with dynamic functional connectivity** | | | | | | | |  |
| **Functional Connectivity** | **VAChT** | **D1** | **DAT** | **M1** | **NET** | **A4B2** | **D2** |  |
| Interactional Dominance | 0.00585 | 0.006633 | 0.004562 | 0.001965 | 0.000021 | 0.00016 | 0.000001 |  |
| Individual Dominance | 0.012741 | 0.008797 | 0.004414 | 0.0004 | 0.000605 | 0.001615 | 0.000713 |  |
| Average Partial Dominance | 0.008409 | 0.006197 | 0.002311 | 0.001951 | 0.000957 | 0.000494 | 0.000618 |  |
| Total Dominance | 0.008662 | 0.006631 | 0.002933 | 0.001732 | 0.000773 | 0.000606 | 0.000543 |  |
| Percentage Relative Importance | 39.590311 | 30.303871 | 13.406233 | 7.91414 | 3.532395 | 2.770719 | 2.482331 |  |

Values represent interactional dominance (shared variance with other predictors), individual dominance (predictor’s isolated contribution), average partial dominance (average incremental contribution across models), total dominance (sum of individual and interactional dominance), and percentage relative importance (proportion of variance explained relative to all predictors). Dominance analysis was performed to assess the contribution of neurotransmitter/gene expression density maps to variability in static and dynamic functional connectivity across 400 cortical regions. Functional connectivity values were estimated using general linear models controlling for age, sex, and education. A_4_B_2_, Alpha-4 Beta-2 Nicotinic Acetylcholine Receptor; B_T_, Participation coefficient; D1, Dopamine D1 Receptor; D2, Dopamine D2 Receptor; DAT, Dopamine Transporter; M1, Muscarinic Acetylcholine Receptor M1; NET, Norepinephrine Transporter; VAChT, Vesicular Acetylcholine Transporter

**Supplementary Table 17. Longitudinal spearmans correlations between functional connectivity changes over time and neurotransmitter densities.**

| **Neurotransmitter correlations with static functional connectivity** | | | | | | | | |
| --- | --- | --- | --- | --- | --- | --- | --- | --- |
| **Functional Connectivity** | **A_4_B_2_** | **M1** | **VAChT** | **D1** | **D2** | **DAT** | **NET** |  |
| observed R statistic | -0.012 | 0.022 | **0.167** | -0.033 | -0.053 | 0.069 | **0.167** |  |
| 2-sided P value | 0.815 | 0.656 | **<0.001** | 0.517 | 0.291 | 0.166 | **<0.001** |  |
| **Neurotransmitter correlations with dynamic between module connectivity** | | | | | | | |  |
| **Participation (B_T_)** | **A_4_B_2_** | **M1** | **VAChT** | **D1** | **D2** | **DAT** | **NET** |  |
| observed R statistic | **0.102** | -0.011 | **0.143** | 0.091 | -0.048 | 0.062 | 0.076 |  |
| 2-sided P value | **0.041** | 0.833 | **0.004** | 0.070 | 0.331 | 0.217 | 0.128 |  |

Values represent the Spearman’s R statistic and two-sided p-value for the correlation between neurotransmitter/gene expression density and changes in functional connectivity across 400 cortical regions. Changes in functional connectivity over time were estimated using linear mixed-effects models controlling for age, sex, and education. Analyses were conducted separately for static functional connectivity and dynamic connectivity (participation coefficient, BT). A_4_B_2_, Alpha-4 Beta-2 Nicotinic Acetylcholine Receptor; B_T_, Participation coefficient; D1, Dopamine D1 Receptor; D2, Dopamine D2 Receptor; DAT, Dopamine Transporter; M1, Muscarinic Acetylcholine Receptor M1; NET, Norepinephrine Transporter; VAChT, Vesicular Acetylcholine Transporter

**Supplementary Table 18. Longitudinal dominance analysis between functional connectivity measures and neurotransmitter densities.**

| **Neurotransmitter dominance with static functional connectivity** | | | | | | | | |
| --- | --- | --- | --- | --- | --- | --- | --- | --- |
| **Functional connectivity** | **VAChT** | **NET** | **D2** | **DAT** | **D1** | **M1** | **A4B2** |  |
| Interactional Dominance | 0.017071 | 0.000311 | 0.023012 | 0.015373 | 0.017553 | 0.019064 | 0.006647 |  |
| Individual Dominance | 0.027875 | 0.027608 | 0.002796 | 0.004811 | 0.001057 | 0.000499 | 0.000138 |  |
| Average Partial Dominance | 0.024991 | 0.017077 | 0.01139 | 0.012322 | 0.011713 | 0.005949 | 0.006194 |  |
| Total Dominance | 0.024272 | 0.016186 | 0.011822 | 0.011685 | 0.011025 | 0.007044 | 0.005394 |  |
| Percentage Relative Importance | 27.761824 | 18.513846 | 13.522255 | 13.365291 | 12.610449 | 8.056804 | 6.16953 |  |
| **Neurotransmitter dominance with dynamic functional connectivity** | | | | | | | |  |
| **Participation (B_T_)** | **VAChT** | **D2** | **D1** | **A4B2** | **DAT** | **NET** | **M1** |  |
| Interactional Dominance | 0.012384 | 0.011674 | 0.003734 | 0.000442 | 0.001122 | 0.000821 | 0.000335 |  |
| Individual Dominance | 0.02053 | 0.002374 | 0.008199 | 0.010431 | 0.003819 | 0.005811 | 0.000112 |  |
| Average Partial Dominance | 0.014575 | 0.010982 | 0.006972 | 0.003816 | 0.002968 | 0.002459 | 0.001559 |  |
| Total Dominance | 0.015113 | 0.009851 | 0.006685 | 0.004279 | 0.002826 | 0.002704 | 0.001178 |  |
| Percentage Relative Importance | 35.44714 | 23.105717 | 15.679343 | 10.035814 | 6.628454 | 6.341682 | 2.761851 |  |

Values represent interactional dominance (shared variance with other predictors), individual dominance (predictor’s isolated contribution), average partial dominance (average incremental contribution across models), total dominance (sum of individual and interactional dominance), and percentage relative importance (proportion of variance explained relative to all predictors). Dominance analysis was performed to assess the contribution of neurotransmitter/gene expression density maps to variability in static and dynamic functional connectivity across 400 cortical regions. Changes in functional connectivity over time were estimated using linear mixed-effects models controlling for age, sex, and education. A_4_B_2_, Alpha-4 Beta-2 Nicotinic Acetylcholine Receptor; B_T_, Participation coefficient; D1, Dopamine D1 Receptor; D2, Dopamine D2 Receptor; DAT, Dopamine Transporter; M1, Muscarinic Acetylcholine Receptor M1; NET, Norepinephrine Transporter; VAChT, Vesicular Acetylcholine Transporter
